# Influence of Charge Compensating Anions on the Adsorption of Perfluorobutanesulfonate in MOF-808

**DOI:** 10.1021/acsomega.5c05815

**Published:** 2025-09-18

**Authors:** Jackson Mikel, Brody Berens, Grace Versnik, Kiley Wadzinski, Trevor Rottiger, Melissa Siewert, Olivia Stellpflug, Shannon C. Riha, Joseph E. Mondloch

**Affiliations:** University of Wisconsin−Stevens Point, Stevens Point, Wisconsin 54481, United States

## Abstract

Per- and poly fluoroalkyl substances (aka PFAS) are a class of anthropogenic compounds that have come under scrutiny given their ability to bioaccumulate in the environment and negatively impact health outcomes. Metal–organic frameworks (MOFs) are an emerging category of sorbents that are attractive for PFAS remediation given their readily modifiable nature. Here we show that the charge compensating anions (cca’s) formate (FA), acetate (AA), trifluoroacetate (TFA), and chloride have a significant impact on the adsorption of perfluorobutanesulfonate (PFBS) within MOF-808. Kinetic measurements indicate that MOF-808 rapidly adsorbs PFBS with equilibrium reached within 50 min or less. The kinetic data is well-fit to the pseudo first-order Langmuir model and the resultant pseudo first-order rate constants vary by a factor of 4 (0.16–0.61 min^–1^) based on the cca’s identity. The adsorption capacity of PFBS also varies by a factor of 4 (95–372 mg/g) when challenged with 500 mg/L solutions and that PFBS adsorption correlates with the quantity of cca’s (monocarboxylate plus chloride) removed during PFBS adsorption. PFBS adsorption isotherms indicate that MOF-808 exhibits excellent maximum adsorption capacities up to 837 mg PFBS/g MOF but binds PFBS relatively weakly (*K*_L_ values no larger than 7.72 × 10^–3^ L/mg). Kinetic, IR spectroscopic, and cca-dependent adsorption data are consistent with PFBS adsorption occurring via ion-exchange of cca’s. Our data demonstrate the importance of controlling and understanding the composition of cca’s when studying PFAS adsorption within MOFs.

## Graphical abstract

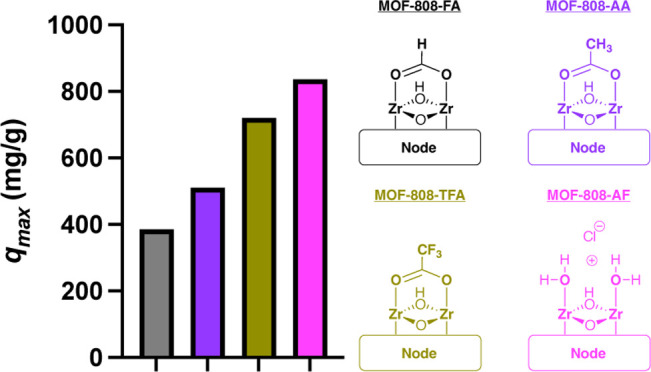

## 1 Introduction

Per- and poly fluoroalkyl substances (aka PFAS or “forever chemicals”) are a large class of man-made substances containing at least one fully fluorinated methyl or methylene carbon.^1^ PFAS have extremely useful properties including high thermal stability, water and stain-resistance, and nonstick behavior.^1^ Unfortunately these same properties ensure that PFAS persist in the environment as well as bioaccumulate in plants,^2^ animals,^3^ and humans.^4^ PFAS can lead to decreased fertility in women, vaccine resistance, developmental effects and/or delays in children, increased cholesterol levels and blood pressure, and increased risk for certain cancers.^5^ Given these concerns, in 2024, the United States Environmental Protection Agency (EPA) set legally enforceable maximum containment levels for the six PFAS molecules shown in Figure 1 to monitor and protect drinking water.^6^

**Figure 1:**
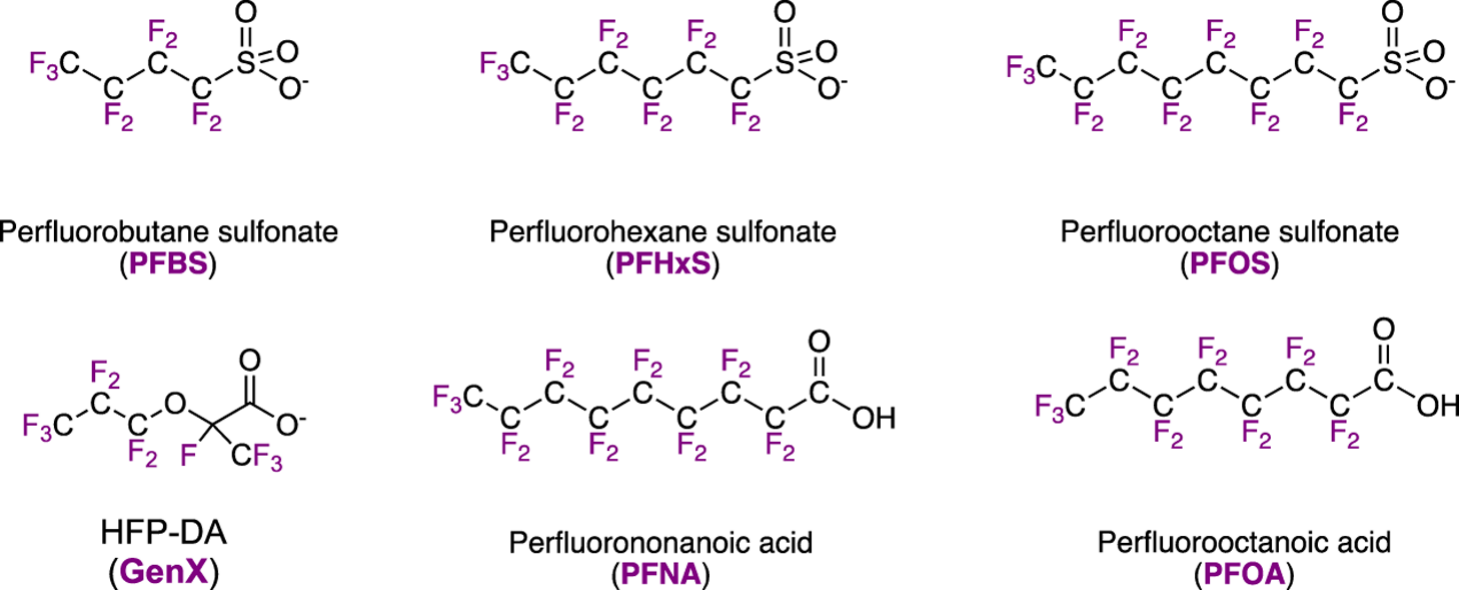
Six important PFAS molecules and their commonly used abbreviations.

Activated carbons and ion-exchange resins are two practical sorption-based technologies utilized to remove PFAS from water.^7^ Unfortunately, these materials can suffer from slow adsorption kinetics, low adsorption capacities (especially for emerging “short-chain” PFAS), reduced performance for PFAS mixtures, and reduced performance in the presence of natural organic matter and other cocontaminants.^8,9^ In water purification settings, these shortcomings require replacement of sorbents on short time scales which are both costly and time-consuming.^10^ There has therefore been ongoing interest in developing novel sorbents that can overcome these challenges.

Metal–organic frameworks (MOFs) are nanoporous solids built up from metal-based nodes and multitopic organic linkers.^11^ MOFs have well-defined and readily modifiable structures, high internal surface areas, and high binding site densities. This makes MOFs attractive candidates for PFAS remediation and recently there has been an interest in evaluating them for this purpose.^8,12,13^ One theme that caught our interest is that MOFs containing [Zr_6_(u_3_-O)_4_(u_3_–OH)_4_]^12+^ nodes (and their closely related derivatives) can effectively remove anionic PFAS, such as perfluoroalkyl acids, via ion exchange of charge compensating hydroxide ions.^14,15^ Experimental evidence for ion-exchange includes a decrease in terminal –OH stretching frequencies post PFAS adsorption,^16^ a single crystal X-ray structure of PFOA bound to [Zr_6_(u_3_-O)_4_(u_3_–OH)_4_]_2_ nodes post adsorption,^15^ and charge compensating anion (cca) dependent PFAS adsorption.^17^ In that study Loukopoulos et al. demonstrated that fluorinated cca’s could enhance PFAS adsorption in two MOFs with [Zr_6_(u_3_-O)_4_(u_3_–OH)_4_]^12+^ nodes.^17^ We wondered if other cca’s—particularly those commonly encountered in the synthesis of MOFs with [Zr_6_(u_3_-O)_4_(u_3_–OH)_4_]^12+^ nodes—might play a role in PFAS adsorption and influence ion-exchange as well.

To test this hypothesis, we turned to the well-known Zr-based MOF, MOF-808, whose formula is often represented as Zr_6_(u_3_-O)_4_(u_3_–OH)_4_(btc)_2_(cca)_6_ (btc = benzenetricarboxylate and cca = a charge compensating anion such as formate).^18,19^ A depiction of some key structural elements of MOF-808 are shown in Figure 2. The node is comprised of six Zr^IV^ ions arranged in an octahedron with each face capped by alternating u_3_-O and u_3_–OH ions. The apical positions of the [Zr_6_(u_3_-O)_4_(u_3_–OH)_4_]^12+^ nodes are linked via 1,3,5-benzenetricarboxylate (btc) linkers. Charge balance is satisfied by six cca’s occupying an equatorial ‘belt’ around the [Zr_6_(u_3_-O)_4_(u_3_–OH)_4_]^12+^ node. Often cca’s are introduced during the synthesis of MOF-808 (and other MOFs containing [Zr_6_(u_3_-O)_4_(u_3_–OH)_4_]^12+^ nodes) and may be monocarboxylate anions (as depicted in Figure 2), pairs of terminal OH^–^/H_2_O ligands, or other anions such as chloride.^16,20^ MOF-808 can be prepared with a host of different cca’s that are known to influence its physical and chemical properties.^16,20,21^

**Figure 2:**
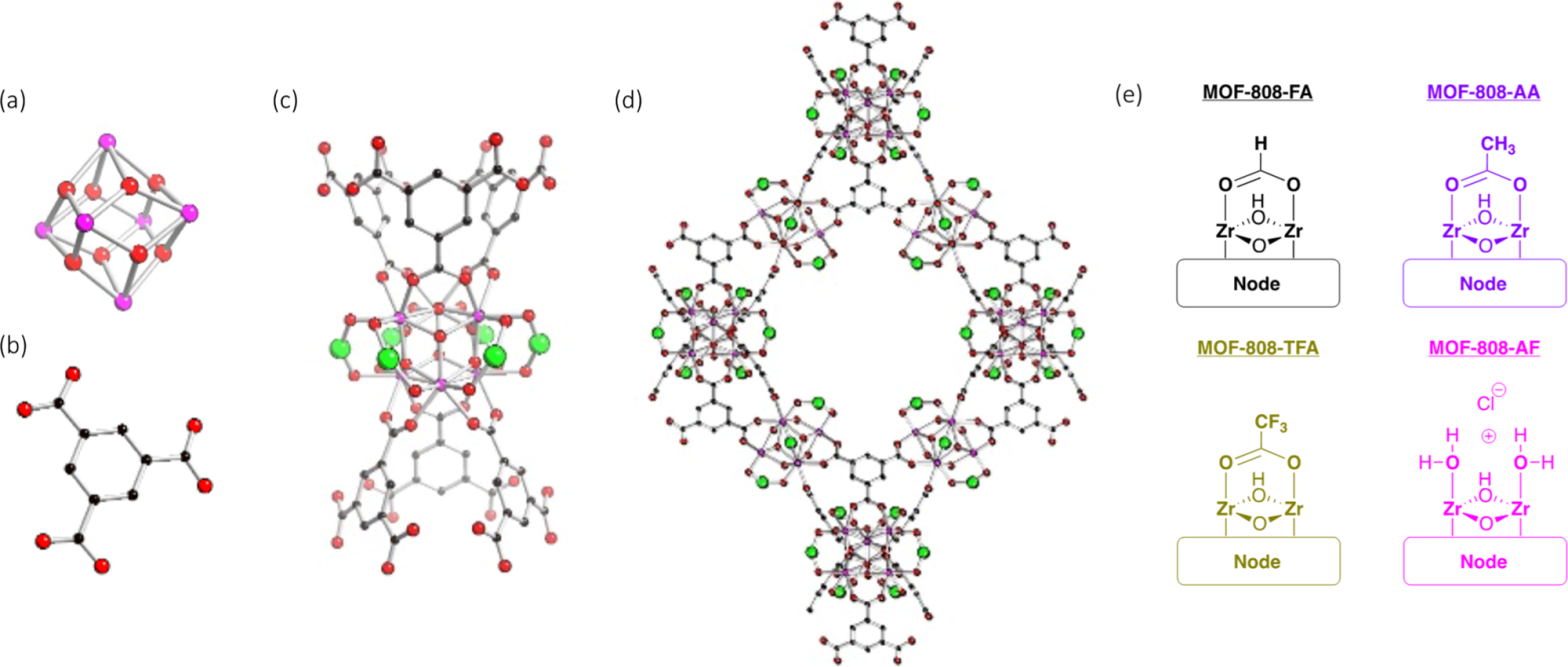
Key structural feature of MOF-808 (H atoms are not shown). (a) The [Zr_6_(u_3_-O)_4_(u_3_–OH)_4_]^12+^ node, (b) the btc linker, (c) connection of btc linkers to the [Zr_6_(u_3_-O)_4_(u_3_–OH)_4_]^12+^ node and the location of carboxylate based cca’s (green spheres), (d) the extended 3-D structure of MOF-808, and (e) representations of the cca’s studied herein.

Here we investigate how the cca’s formate, acetate, trifluoroacetate, and chloride (shown in Figure 2e)—which were incorporated during the synthesis of MOF-808—influence the adsorption of PFBS. Pseudo first order rate constants and adsorption capacities vary by a factor of approximately four based on the identity of the cca’s present. NMR and EDX analysis indicate that both monocarboxylate and chloride cca’s can be present and that PFBS uptake correlates with the amount of cca’s removed from the [Zr_6_(u_3_-O)_4_(u_3_–OH)_4_]^12+^ nodes. The demonstration that inorganic anions such as chloride can undergo ion-exchange in MOF-808 is important for the development of practical sorbents as the release of many monocarboxylates (e.g., formate and trifluoroacetate) into waste streams is less than desirable. PFBS isotherms for all MOF-808 derivatives were well-fit to the Langmuir adsorption model and adsorption capacities up to 837 mg/g were observed. This is the highest known adsorption capacity for PFBS within MOFs to date. The cca-dependent adsorption data along with kinetic and IR spectroscopic data are consistent with ion-exchange occurring between PFBS and cca’s within MOF-808. The extracted Langmuir parameters (*K*_L_ values no larger than 7.72 × 10^–3^ L/mg) from the PFBS adsorption isotherms indicate relatively weak interactions between PFBS and MOF-808 hinting that ion-exchange occurs through an outer-sphere pathway.

## 2 Methods

### 2.1 Materials

Zirconyl chloride octahydrate (98%), 1,3,5-benzenetricarboxylic acid (98%), formic acid (98+%), acetic acid (99.5%), trifluoroacetic acid (99%), potassium phosphate tribasic (97%), potassium nonafluoro-1-butanesulfonate (98%), 2,6-bis­(trifluoromethyl) benzoic acid (98%), concentrated hydrochloric acid (37.5%), ethanol (reagent grade), and deuterium oxide (D, 99.9%) were purchased from commercial vendors and used without further purification. Powder X-ray diffraction (PXRD) data was collected at room temperature on a Bruker Advance Eco powder diffractometer with a ceramic Cu tube (CuKα radiation, λ = 1.54 Å) and a 1-D solid-state silicon strip detector. The source was operated at 20 kV/5 mA (100 W). Data was collected from 3 to 55 two-theta with a 1 s step. Nitrogen gas adsorption isotherms were collected at 77 K on a Micromeritics TriStar II plus. The samples were activated on a Micromeritics VacPrep 061 at 110 °C under dynamic vacuum for 18 h. Approximately 50 mg of sample was utilized for each measurement. ^1^H and ^19^F­{^1^H} NMR spectra were collected on a Bruker Avance III HD 400 MHz spectrometer equipped with a Sample Express autosampler. Attenuated total reflection (ATR) infrared spectroscopy data from 500–4000 cm^–1^ were collected on a Thermo Nicolet iS5 spectrometer with an iD7 ATR accessory. SEM–EDX data were collected on a Hitachi S-3400N–II scanning electron microscopy with a thermo energy-dispersive X-ray spectroscopy (EDX) detector. The accelerating voltage for EDX analysis was 25 kV and five spectra for each sample were collected and averaged to determine the amount of chloride and zirconium. Thermogravimetric analysis (TGA) traces were collected from room temperature to 900 °C under compressed air on a TA Instruments SDT-650 utilizing a ramp rate of 10 °C/min.

### 2.2 Synthesis of MOF-808 Derivatives

#### 2.2.1 MOF-808-FA

966 mg (3.0 mmol) of ZrOCl_2_·8H_2_O and 210 mg (1 mmol) of H_3_BTC were weighed (using filter paper) and added to a 50 mL round-bottom flask. Subsequently 10 mL of DI H_2_O, 10 mL of formic acid, and 100 μL of concentrated HCl were added (in that order) along with a stir bar. The solution was refluxed at 110 °C for 18 h while stirring at 600 rpm. The resultant white powder was collected by centrifugation and washed/centrifuged 3× with 20 mL of DI water and 3× with 20 mL of ethanol while waiting at least 10 min in between washings. After the last wash the powder was dried in an oven at 80 °C for 18 h. MOF samples were stored in a desiccator in between use.

#### 2.2.2 MOF-808-AA

966 mg (3.0 mmol) of ZrOCl_2_·8H_2_O and 210 mg (1 mmol) of H_3_BTC were weighed (using filter paper) and added to a 50 mL round-bottom flask. Subsequently 10 mL of DI H_2_O, 10 mL of acetic acid, and 100 μL of concentrated HCl were added (in that order) along with a stir bar. The solution was refluxed at 110 °C for 18 h while stirring at 600 rpm. The resultant white powder was collected by centrifugation and washed/centrifuged 3× with 20 mL of DI water and 3× with 20 mL of ethanol while waiting at least 10 min in between washings. After the last wash the powder was dried in an oven at 80 °C for 18 h. MOF samples were stored in a desiccator in between use.

#### 2.2.3 MOF-808-TFA

966 mg (3.0 mmol) of ZrOCl_2_·8H_2_O and 210 mg (1 mmol) of H_3_BTC were weighed (using filter paper) and added to a 50 mL round-bottom flask. Subsequently 10 mL of DI H_2_O and 5 mL of trifluoroacetic acid were added to the 50 mL round-bottom flask (in that order) along with a stir bar. The solution was refluxed at 120 °C for 18 h while stirring at 600 rpm. The resultant white powder was collected by centrifugation and washed/centrifuged 6× with 25 mL of DI water over the course of 1 day and 6× with 25 mL of ethanol over the course of 1 day. After the last wash the powder was dried in an oven at 80 °C for 18 h. MOF samples were stored in a desiccator in between use.

#### 2.2.4 MOF-808-AF

MOF-808-AA was synthesized as described above. The resultant white slurry was transferred into a two 50 mL conical bottom centrifuge tubes and washed/centrifuged/decanted (centrifugation = 8500 rpm for 2 min) three times with 25 mL of DI water over the course of a day. The last solution was allowed to sit overnight prior to decanting. After the third decant 15 mL of 1 M HCl was added to the conical bottom tube and the mixture was shaken at 500 rpm and 85 °C. Over the course of 8 h the solution was cooled, centrifuged, and the 1 M HCl was replaced three times. The resultant powder was washed/centrifuged/decanted three times with 25 mL of DI waster and three times with acetone over the course of 24 h for each solvent. After the last wash/centrifuge/decant step the solids were placed in an oven at 80 °C under vacuum.

### 2.3 PFBS Adsorption

In triplicate, approximately 10 mg of MOF-808 was weighed into separate 15 mL polypropylene centrifuge tubes. One mL of 500 mg/L PFBS per mg of MOF was added to the same tube. The tubes were capped and placed in a temperature-controlled shaker at 25 °C and 300 rpm until equilibrium was reached as judged by kinetic traces (vide infra). The resultant solutions were centrifuged at 4900 rpm for 2 min and then 700 μL of the solution was transferred into an NMR tube. 50 μL of an 8.29 mM 2,6-TFMBA (added as an internal standard) solution in D_2_O was added to the NMR tube. The tube was capped and inverted at least five times to ensure homogeneous mixing. As previously demonstrated,^22^ quantitative ^19^F­{^1^H} NMR spectra were collected using no spinning, a relaxation delay of 20 s, a spectral width of 40 ppm, and an O1P of −70 ppm. T_1_ relaxation measurements and their associated fits are shown in the Supporting Information (Figure S1 and Table S1). ^1^H NMR spectra were collected using no spinning and a relaxation delay of 20 s.

The initial concentration of PFBS in the NMR tube was calculated using eq 1 and converted into experimental concentrations using the dilution equation. Here *I*_PFBS_ and *I*_2,6‑TFMBA_ are integrals of PFBS and 2,6-TFMBA (the internal standard), and *N*_PFBS_ and *N*_2,6‑TFMBA_ represents the number of fluorine atoms corresponding to the signal that was integrated in the NMR spectrum (i.e., three for PFBS due to the terminal –CF_3_ group on PFBS and 6 for 2,6-TFMBA).

1 $${\left[PFBS\right]}_{NMRtube}=\frac{I_{PFBS}}{I_{2,6‐TFMBA}}\times \frac{N_{2,6‐TFMBA}}{N_{PFBS}}\times {\left[2,6‐TFMBA\right]}_{NMRtube}$$

Control experiments were performed in the absence of MOF to determine the starting concentration of the PFBS solution. Adsorption capacities (*q*, mg PFAS/g MOF) were calculated using eq 2 where *C*_0_ is the initial PFBS concentration in solution, *C* is the PFBS concentration after equilibrium was reached, *V* is the volume of the solution (in L), and *m* was the mass of the sorbent (in g).

2 $$q=\frac{\left(C_{0}-C\right)\times V}{m}$$

### 2.4 Characterization of CCA’s

#### 2.4.1 Pre PFBS Adsorption

The quantity of monocarboxylate cca’s (FA, AA, or TFA) was determined utilizing the following procedures. 250 mg of potassium phosphate tribasic (K_3_PO_4_) was weighed into a high recovery vial along with a spin-vane stir bar. 1.00 mL of deuterium oxide (D_2_O) for MOF-808-FA or MOF-808-AA or 1.00 mL of water (H_2_O) for MOF-808-TFA was added to the vial and the solution was mixed until K_3_PO_4_ dissolved. Approximately 1 mg of MOF-808 was added to the vial and the mixture was stirred at 1000 rpm until the MOF was completely digested. For MOF-808-FA and MOF-808-AA 700 μL of the digestion solution was transferred to an NMR tube and a ^1^H NMR spectrum was collected using a relaxation delay of 20 s without sample spinning. For MOF-808-TFA 600 μL of the digestion solution and 100 μL of 8.29 mM 2,6-bis­(trifluoromethyl)­benzoic acid (2,6-TFMBA) in D_2_O was added to the NMR tube. The NMR tube was capped and inverted at least five times and ^1^H and/or ^19^F­{^1^H} spectra were collected. Zr and Cl content was determined using SEM–EDX analysis. The as dried samples were deposited on carbon tape and a minimum five EDX spectra were collected for each sample, from which the data was averaged to determine the mole ratio.

#### 2.4.2 Post PFBS Adsorption

Post-PFBS adsorption solutions were centrifuged at 4900 rpm for 2 min and the PFBS solution was decanted. The solid was washed 3× with 15 mL of acetone (via centrifugation and decanting) and dried in an oven at 80 °C for 18 h. Dried solids were analyzed as described in the Pre PFBS Adsorption section by PXRD, ATR IR spectroscopy, ^1^H and/or ^19^F­{^1^H} NMR (potassium phosphate digested solutions), and SEM–EDX analysis.

### 2.5 PFBS Kinetic Measurements

Approximately 40 mg of MOF-808 was weighed into a 50 mL polypropylene centrifuge tube. One mL of approximately 450 mg/L PFBS per mg of MOF was added to the tube. The tubes were capped and placed in a shaker at 25 °C and shaken at 1000 rpm until equilibrium was reached. At each time point the solution was centrifuged at 4900 rpm for 2 min and subsequently 1 mL of solution was filtered through a 0.22 μm PTFE syringe filter. 500 μL of the filtered solution was transferred into an NMR tube and a coaxial insert containing 50 μL of 8.29 mM 2,6-TFMBA in D_2_O was added to the NMR tube. For each data point quantitative ^19^F­{^1^H} NMR spectra were collected using no spinning, a relaxation delay of 20 s, a spectral width of 40 ppm, and an O1P of −70 ppm. PFBS adsorption values (*q*_e_, mg/g) were calculated utilizing eqs 1 and 2. Time-dependent adsorption data was fit to the pseudo first-order Langmuir eq (eq 3) where *q*_*t*_ is the adsorption capacity at time *t*, *q*_e_ is the adsorption capacity at equilibrium, *k*_1_ is the pseudo first-order rate constant, and *t* is time.

3 $$q_{t}=q_{e}\left(1-e^{-k_{1}t}\right)$$

### 2.6 PFBS Adsorption Isotherms

Approximately 10 mg of MOF-808 was weighed into a 15 mL polypropylene centrifuge tube. One mL of variable concentration PFBS (e.g., 100 mg/L–2500 mg/L) per mg of MOF was added to the same tube. The tubes were capped and shaken at 300 rpm and 25 °C for 2 h (i.e., until a time when equilibrium was reached). The solutions were centrifuged at 4900 rpm for 2 min and 700 μL of the solution was transferred into an NMR tube. 50 μL of 8.29 mM 2,6-TFMBA in D_2_O was added to the NMR tube, a cap was added, and the solutions were inverted at least five times to ensure homogeneous mixing. Quantitative ^19^F­{^1^H} NMR spectra were collected and PFBS adsorption values (*q*_e_, mg/g) were calculated utilizing eqs 1 and 2. PFBS adsorption isotherms were fit to the Langmuir (eq 4) and Freundlich eqs (eq 5). In equations four and five *q*_e_ is the equilibrium quantity of PFBS in the sorbent and *c*_e_ is the equilibrium concentration of PFBS in solution. In the Langmuir equation *q*_max_ (mg/g) is the maximum adsorption capacity, while *K*_L_ is the Langmuir constant (L/mg) which indicates the strength of interaction between the adsorbate and sorbent. In the Freundlich equation *K*_f_ is the Freundlich constant (i.e., the maximum adsorption capacity) and *n* is a dimensionless factor that describes the heterogeneity of the adsorption surface.

4 $$q_{e}=\frac{q_{\max}K_{L}c_{e}}{1+K_{L}c_{e}}$$

5 $$q_{e}=K_{f}c_{e}^{1/n}$$

## 3 Results

### 3.1 Synthesis and Characterization of MOF-808 Derivatives

MOF-808-FA, -AA, and -TFA were synthesized utilizing a slightly modified procedure from Liu et al.,^16^ while MOF-808-AF was synthesized using a modified procedure from Lyu et al.^19^ All four MOF-808 derivatives were crystalline and have surface areas (2133, 1875, 1461, and 1808 m^2^/g for MOF-808 FA, -AA, -TFA, and -AF) and pore volumes (0.85, 0.79, 0.61, and 0.76 cm^3^/g for MOF-808 FA, -AA, -TFA, and -AF) as detailed in the Supporting Information (Figures S2 and S3 and Table S2).

To gain an understanding of the role that cca’s play in PFBS adsorption we identified and quantified the cca’s in each MOF-808 derivative under our synthetic conditions. Example data for MOF-808-FA, which contains both monocarboxylates and chloride, is shown in Figure 3 and the results are summarized in Table 1. Some of the other MOF-808 derivatives do not contain both types of cca’s as summarized in Table 1 and the raw data is shown in the Supporting Information. The quantity of monocarboxylate cca’s was determined using ^1^H and/or ^19^F­{^1^H} NMR spectroscopy from potassium phosphate digested samples (Figures S4–S13). MOF-808-FA and MOF-808-AA contain 4.0 eq of monocarboxylate cca’s, while MOF-808-TFA contains 5.2 eq of monocarboxylate per [Zr_6_(u_3_-O)_4_(u_3_–OH)_4_]^12+^ node. Treatment of MOF-808-AA with 1 M HCl nearly quantitatively removes the acetate ligands (0.2 eq remaining) and leaves behind 3.8 eq of Cl^–^ per [Zr_6_(u_3_-O)_4_(u_3_–OH)_4_]^12+^ node as quantified by SEM–EDX (Figure S14). Under our synthetic conditions there was also 0.8, 1.6, and 0 eq of chloride per [Zr_6_(u_3_-O)_4_(u_3_–OH)_4_]^12+^ node for MOF-808-FA, -AA, and -TFA.

**Figure 3:**
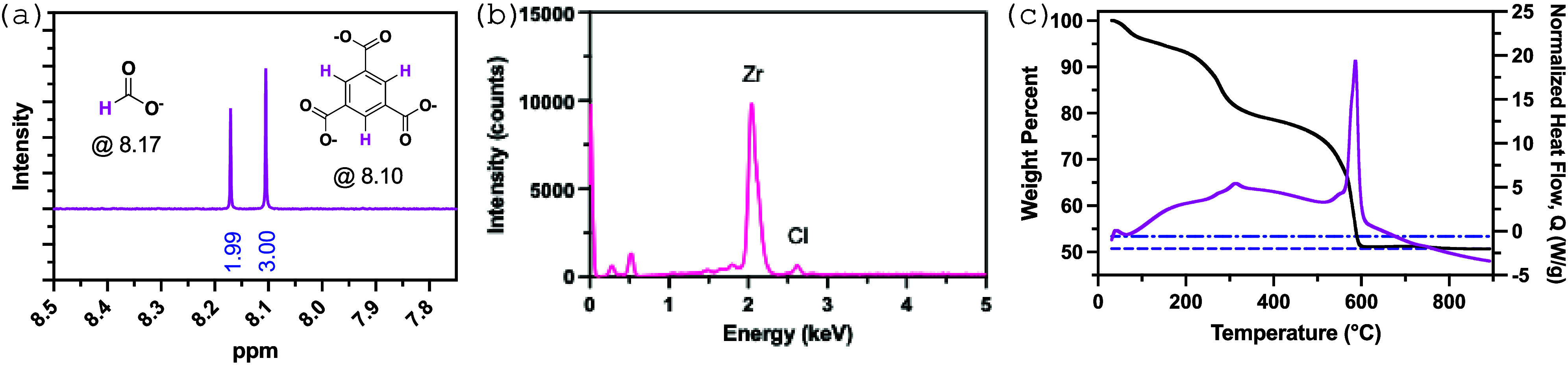
Representative examples of (a) ^1^H NMR for monocarboxylate determination, (b) EDX for chloride determination, and (c) TGA (black) and DTA (pink) curve for water determination in MOF-808-FA. The dashed blue line indicates the expected weight percent with no water, while the dashed purple line indicates the expected weight percent with water incorporated into the formula weight.

**Table 1:** Summary of cca’s and EtOH Found per [Zr_6_(u_3_-O)_4_(u_3_–OH)_4_]^12+^ Node in Each MOF-808 Derivative Prior to PFBS Adsorption

MOF-808-	carboxylate	chloride	EtOH
FA	4.0	0.8	1.8
AA	4.0	1.6	2.5
TFA	5.2	0.0	2.0
AF	0.2	3.8	0.0

The carboxylate and chloride data in Table 1 assume that linker defects are not present at appreciable quantities in MOF-808 (i.e., there should be two btc linkers per [Zr_6_(u_3_-O)_4_(u_3_–OH)_4_]^12+^ node). To corroborate this assumption, we turned to thermal gravimetric analysis (TGA) which has been used extensively to probe for missing linker defects in MOFs containing [Zr_6_(u_3_-O)_4_(u_3_–OH)_4_]^12+^ nodes.^23^ When missing linkers are present the final weight percent in the TGA curve (i.e., that which corresponds to the formation of 6 eq of ZrO_2_ after heating MOF-808) will be higher than theoretically expected based on the formula mass of the MOF. However, for this series of MOF-808 derivatives we see weight percentages that are lower than expected, indicating that missing linker defects are not present. Some of the unaccounted-for mass comes from EtOH, which was used to wash our MOFs after synthesis. ^1^H NMR data indicates 1.8, 2.5, 2.0, and 0 equiv of ethanol per btc linker in MOF-808-FA, -AA, -TFA, and -AF (Table 1).

The expected final weight percents when considering the monocarboxylate, chloride, and ethanol is shown as the dashed blue line in Figure 3c (the remaining MOF-808 derivatives shown in Figure S15). We’ve assigned the remaining unaccounted-for mass to water molecules, four for MOF-808-FA and -AA, six for MOF-808-TFA, and twenty-six for MOF-808-AF. When included the final observed and theoretical weight percents (dashed purple line, Figures 3c and S15) are in excellent agreement. The inclusion of H_2_O molecules in our formula units are consistent with the presence of pore bound water (broad peaks centered at approximately 3300 cm^–1^) observed in our IR spectra as shown in Figure S16.^24^ The resultant formulas for MOF-808-FA, -AA, -TFA, and -AF are then Zr_6_(u_3_-O)_4_(u_3_–OH)_4_(btc)_2_ (FA)_4_Cl_0.8_(C_2_H_5_O)_1.8_(H_2_O)_4_, Zr_6_(u_3_-O)_4_(u_3_–OH)_4_(btc)_2_(AA)_4_ Cl_1.6_(C_2_H_5_O)_2.5_(H_2_O)_4_, Zr_6_(u_3_-O)_4_(u_3_–OH)_4_(btc)_2_(TFA)_5.2_ (C_2_H_5_O)_2.0_(H_2_O)_4_, Zr_6_(u_3_-O)_4_(u_3_–OH)_4_(btc)_2_(AA)_0.2_ Cl_3.8_(H_2_O)_26_.

The astute reader will recognize that there should be six cca’s per [Zr_6_(u_3_-O)_4_(u_3_–OH)_4_]^12+^ node in MOF-808 but the data in Table 1 indicate less than six cca’s for each derivative. Often in MOFs with [Zr_6_(u_3_-O)_4_(u_3_–OH)_4_]^12+^ nodes the remaining charge compensation is assigned as hydroxide/aqua (OH^–^/H_2_O) ligand pairs. This is the most likely possibility for MOF-808, but it is also possible that charge compensation is satisfied by ethoxide/ethanol (EtO^–^/EtOH) ligand pairs from synthesis. This could explain the fact that EtOH is not removed under our thermal activation conditions (80 °C) as terminal hydroxides on [Zr_6_(u_3_-O)_4_(u_3_–OH)_4_]^12+^ nodes are known to react with alcohols at room temperature and leave behind surface bound alkoxides.^21,25^ However, guest molecules could also bind strongly to [Zr_6_(u_3_-O)_4_(u_3_–OH)_4_]^12+^ nodes via H-bonding. In short, charge compensation must come from either –OH^–^/H_2_O or EtO^–^/EtOH pairs, but our analysis is unable to distinguish between the two.

To see if cca’s impact PFBS adsorption we weighed approximately 10 mg of MOF-808-FA, -AA, -TFA, or -AF into 15 mL polypropylene centrifuge tubes. One mL of 500 mg/L PFBS solution per mg of MOF was subsequently added to the tube. We will subsequently refer to this as a standard conditions reaction. The amount of PFBS in solution before and after adsorption was measured via quantitative ^19^F­{^1^H} NMR spectroscopy using 2,6-trifluoromethylbenzoic acid (2,6-TFMBA) as an internal standard. T_1_ relaxation measurements to ensure quantitative recovery of the NMR signal between pulses are shown in Figures S1 and Table S1. Example ^19^F­{^1^H} NMR spectra pre and post PFBS adsorption for MOF-808-FA (black line), -AA (purple line), -TFA (gold line), and -AF (pink line) are shown in Figure 4a. Also shown in Figure 4a is a spectrum of the 500 mg/L starting solution (blue line). The resonances at 59.73 ppm are assigned to the –CF_3_ groups of the 2,6-TFMBA internal standard while those centered at 80.85 ppm are the terminal –CF_3_ group of PFBS. The signal centered at 75.48 ppm in the MOF-808-TFA spectrum is TFA. We used the resonances at 59.73 and 80.85 ppm from triplicate measurements along with eqs 1 and 2 to calculate the average PFBS adsorption capacity for each MOF-808 derivative. As shown in Figure 4b, PFBS adsorption under these conditions varies by a factor of approximately four, from 95(4) mg/g for MOF-808-FA (black), to 139(4) mg/g for MOF-808-AA (purple), to 263(4) mg/g for MOF-808-TFA (gold), and 372(14) for MOF-808-AF (pink).

**Figure 4:**
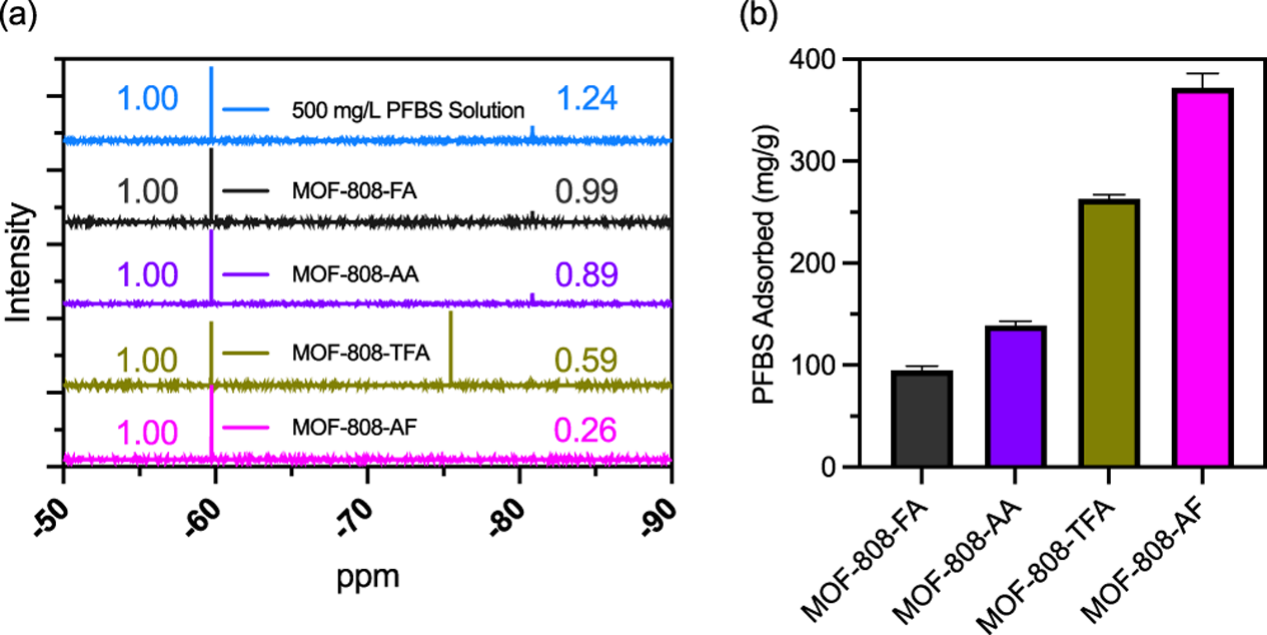
(a) Example ^19^F­{^1^H} NMR spectra for a 500 mg/L PFBS solution (pink) and after PFBS adsorption (black, purple, gold). (b) Average PFBS adsorption capacities after reaching equilibrium in contact with 500 mg/L PFBS solutions.

### 3.2 Post PFBS Adsorption Characterization

There were no changes in the MOF-808 PXRD patterns post PFBS adsorption (Figure S17) confirming the structural integrity of the framework was maintained under our standard conditions. Attenuated total reflection (ATR) infrared spectroscopy indicated the presence of PFBS loaded onto each MOF-808 derivative. The ATR data is shown in Figure 5 where the black lines indicate the pure MOF-808 derivatives and the pink lines show the PFBS loaded frameworks. After PFBS adsorption three new bands appeared at approximately 1240, 1138, and 1064 cm^–1^ which are consistent with the symmetric and asymmetric stretching of –CF_2_ and –SO_3_^–^ respectively.^26^ Further inspection of the ATR spectra of MOF-808-TFA indicates significant loss of the bands at 1660 and 1159 cm^–1^ which are assigned to the asymmetric –CO_2_ and –CF_3_ stretching of TFA.^27^

**Figure 5:**
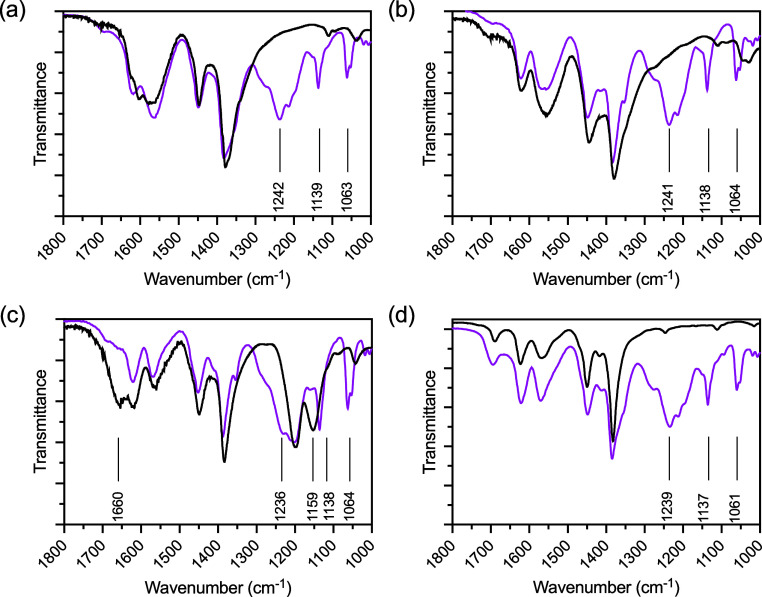
ATR IR of (a) MOF-808-FA (b) MOF-808-AA, (c) MOF-808-TFA, and (d) MOF-808-AF. The black lines are the parent materials and the pink lines are loaded with PFBS.

Next, we turned out attention to quantifying the monocarboxylate and chloride cca’s in each MOF-808 derivative after PFBS adsorption under our standard conditions. Table 2 shows the quantity of monocarboxylate cca’s (FA, AA, or TFA) and chloride per [Zr_6_(u_3_-O)_4_(u_3_–OH)_4_]^12+^ node after PFBS adsorption. The quantity of monocarboxylate cca’s was determined using ^1^H and ^19^F­{^1^H} NMR spectroscopy from potassium phosphate digested samples (Figures S4–S13), while the quantity of chloride was determined using SEM–EDX. After PFBS adsorption the amount of monocarboxylate cca decreases to 3.5, 3.2, 2.7, and 0 eq per [Zr_6_(u_3_-O)_4_(u_3_–OH)_4_]^12+^ node for MOF-808-FA, -AA, -TFA, and -AF. The quantity of chloride also decreases to 0.1, 0.6, and 1.0 eq per [Zr_6_(u_3_-O)_4_(u_3_–OH)_4_]^12+^ node for MOF-808-FA, -AA, and -AF (recall there was no chloride present in MOF-808-TFA pre-PFBS adsorption). There was no EtOH left after PFBS adsorption for any MOF-808 derivative as shown in Figures S4–S13. Control experiments carried out with each MOF-808 derivative in the absence of PFBS (i.e., in just DI water) reveal background dissociation of cca’s as well as ethanol. That data is shown in Table S4 and Figures S19–23.

**Table 2:** Summary of cca’s and EtOH Found in Each MOF-808 Derivative after PFBS Adsorption

MOF-808-	carboxylate	chloride	EtOH
FA	3.5	0.1	0.0
AA	3.2	0.6	0.0
TFA	2.7	0.0	0.0
AF	0.0	1.0	0.0

#### 3.2.1 Kinetic Measurements

To further assess PFBS adsorption we carried out kinetic measurements. PFBS kinetic data for MOF-808-FA (black squares), MOF-808-AA (purple circles), MOF-808-TFA (yellow triangles), and MOF-808-AF (pink) are shown in Figure 6. The solid lines are nonlinear curve fits to the pseudo first-order Langmuir eq (eq 3) which fit the data well (*R* ^2^ > 0.99 for each MOF-808 derivative, Table 2). PFBS adsorption is rapid, reaching equilibrium within 50 min for each MOF-808 derivative. While the pseudo first order rate constants (*k*_1_) vary by a factor of approximately four (Table 3), the rapid uptake of PFBS makes it difficult to determine if cca’s are indeed influencing the adsorption kinetics. This is reflected by the dotted lines and ranges for *k*_1_ in Table 3 which indicate the 95% confidence interval parameters from each curve fit.

**Figure 6:**
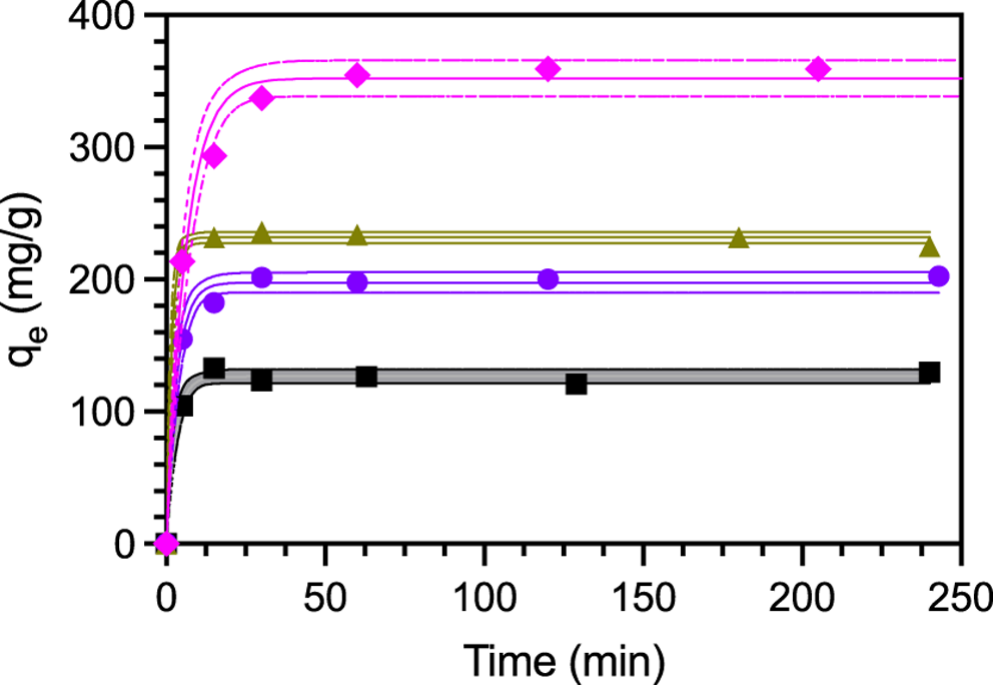
Kinetic measurements of PFBS adsorption in MOF-808-FA (black), -AA (purple), -TFA (gold), and -AF (pink). The solid lines are the best fit line to the pseudo first-order Langmuir equation and the dotted lines represent the 95% confidence interval for that best fit.

**Table 3:** Summary Pseudo First Order Rate Constants and *R* ^2^ Values for Fits to the Pseudo First-Order Langmuir Equation (Eq 3)

MOF-808-	*k*_1_ (min^–1^)	*k*_1_ 95% CI	*R* ^2^
FA	0.35	0.266–0.520	0.993
AA	0.30	0.227–0.407	0.994
TFA	0.61	0.479–1.231	0.999
AF	0.16	0.124–0.216	0.988

### 3.3 PFBS Adsorption Isotherms

PFBS adsorption isotherms were collected to gain a deeper understanding into the adsorption capacity and binding interactions of PFBS with each MOF-808 derivative. Figure 7 shows PFBS adsorption isotherms for MOF-808-FA (black circles), MOF-808-AA (purple squares), MOF-808-TFA (gold triangles), and MOF-808-AF (pink hexagons). The solid lines in Figure 7 are fits to the Langmuir eq (eq 4), while the dashed lines and areas between them indicate 95% confidence intervals from those fits. Fits to the Freundlich eq (eq 5) were inferior for all MOF-808 derivatives (Figure S18 and Table S4). The extracted fitting parameters are shown in Table 4. The maximum PFBS adsorption capacities (*q*_max_) are 385, 510, 721, and 832 mg/g for MOF-808-FA, -AA, -TFA, and -AF, which mirror that observed for PFBS adsorption starting from 500 mg/L solutions under our standard conditions (i.e., Figure 3b). The Langmuir constants (*K*_*L*_) are also summarized in Table 4 and are 1.32 × 10^–3^, 4.15 × 10^–3^, 3.27 × 10^–3^, and 7.72 × 10^–3^ for MOF-808-FA, -AA, -TFA, and -AF respectively.

**Figure 7:**
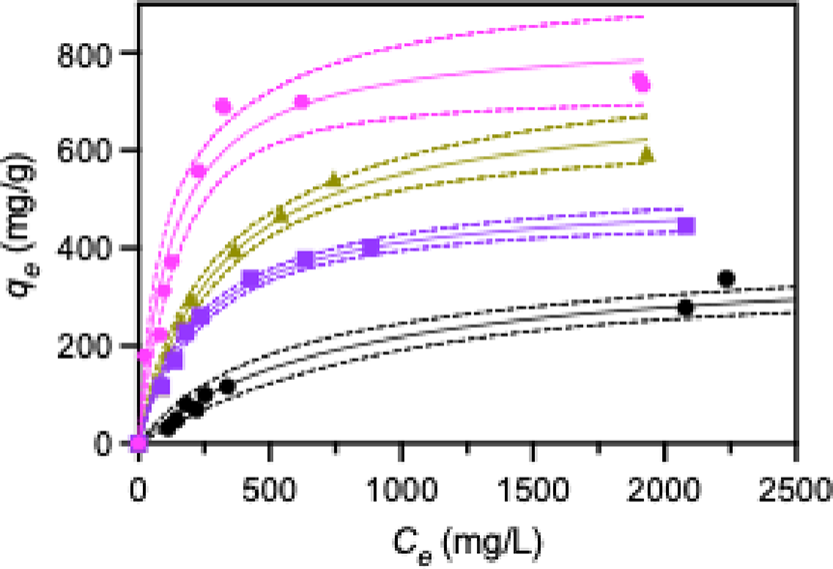
PFBS adsorption isotherms for MOF-808-FA (black), -AA (purple), -TFA (gold), and -AF (pink). The solid lines are the best fit line to the Langmuir isotherm model, while the dotted lines represent the 95% confidence interval for that best fit.

**Table 4:** Summary of PFBS Isotherm Fitting Parameters

MOF	*q*_max_ (mg/g)	*q*_max_ 95% CI	*K*_L_ (L/mg)	*K*_L_ 95% CI	Langmuir *R* ^2^	Freundlich *R* ^2^
MOF-808-FA	385	334–450	1.32 × 10^–3^	8.02 × 10^–4^ to 2.04 × 10^–3^	0.966	0.906
MOF-808-AA	511	478–546	4.15 × 10^–3^	3.42 × 10^–3^ to 5.05 × 10^–3^	0.993	0.931
MOF-808-TFA	721	649–802	3.27 × 10^–3^	2.50 × 10^–3^ to 4.30 × 10^–3^	0.987	0.917
MOF-808-AF	837	732–954	7.72 × 10^–3^	5.01 × 10^–3^ to 1.25 × 10^–2^	0.945	0.832

## 4 Discussion

### 4.1 Effect of Charge Compensating Anions

Our original hypothesis was that cca’s commonly used in the synthesis of MOFs with [Zr_6_(u_3_-O)_4_(u_3_–OH)_4_]^12+^ nodes would influence the adsorption of PFBS within MOF-808. The cca-dependent kinetic and adsorption data shown in Figures 4, 6, and 7 clearly indicate that cca’s affect PFBS adsorption within MOF-808. Our results are qualitatively consistent with those of with Loukopoulos et al.’s who found that formate and trifluoroacetate influence the amount of perfluorobutanoic and perfluorooctanoic acid adsorbed in MOF-808.^17^ Comparison of Tables 1 and 2 indicate that the quantity of cca’s decreases after PFBS adsorption. The background subtracted difference (i.e., subtraction of columns in Table S4 from those in Table 2) in monocarboxylate and chloride, before and after PFBS adsorption, for each MOF-808 derivative are summarized in Table 5. For example, the amount of formate in MOF-808-FA decreases by 0.2 eq per [Zr_6_(u_3_-O)_4_(u_3_–OH)_4_]^12+^ node, while the amount of chloride decreases by 0.3 eq per [Zr_6_(u_3_-O)_4_(u_3_–OH)_4_]^12+^ node. The total loss of cca’s decreases by 0.5 eq per [Zr_6_(u_3_-O)_4_(u_3_–OH)_4_]^12+^ node. We’ve found that the amount of PFBS adsorbed (i.e., the data from Figure 4b) correlates with the total quantity of monocarboxylate plus chloride cca’s removed (i.e., column two in Table 5) from the MOF-808 nodes as shown in Figure 8a. To further investigate the role of monocarboxylate and chloride on PFBS adsorption we plotted equivalents of monocarboxylate and chloride removed vs PFBS adsorption in Figure 8b,c. Both correlate with PFBS adsorption when MOF-808-AF is ignored in Figure 8b and MOF-808-TFA is ignored in Figure 8c. This is reasonable as MOF-808-AF contains very little monocarboxylate and MOF-808-TFA contains no detectable chloride. The results suggest that both monocarboxylate and chloride play a role in PFBS adsorption in MOF-808.

**Table 5:** Difference in Monocarboxylate and Chloride cca’s before and after PFBS Adsorption

MOF-808-	change in carboxylate + chloride	change in carboxylate	change in chloride
FA	0.5	0.2	0.3
AA	1.2	0.6	0.6
TFA	1.3	1.3	0.0
AF	1.9	0.1	1.8

**Figure 8:**
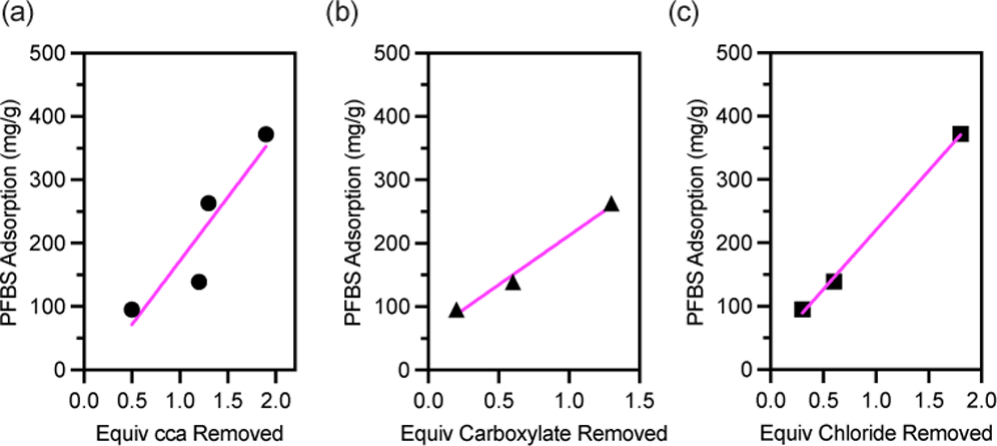
PFBS adsorption vs equivalents of (a) total cca’s removed, (b) monocarboxylate cca’s removed, and (c) chloride cca’s removed from the [Zr_6_(u_3_-O)_4_(u_3_–OH)_4_]^12+^ nodes.

### 4.2 Ion Exchange

The cca dependent adsorption data shown in Figures 4 and 7 coupled with the empirical dependence of PFBS adsorption on the loss of cca’s from the MOF-808 framework shown in Figure 8 is consistent with PFBS adsorption occurring through an ion-exchange mechanism. The fact that monocarboxylate cca’s can undergo ion exchange is consistent with the data reported by Loukopoulos et al.,^17^ but our results highlight that acetate and chloride can also undergo ion-exchange within MOF-808. We suspect this insight will be important for the development of practical sorbents as many monocarboxylates (e.g., formate and trifluoroacetate) are less than desirable byproducts from ion-exchange resins.

Additional evidence for ion exchange comes from a closer look at MOF-808-TFA. Inspection of the ATR data in Figure 5c indicates the loss of stretches assigned to TFA at 1660 and 1159 cm^–1^ and the appearance of stretches assigned to PFBS at 1240, 1138, and 1064 cm^–1^, data consistent with ion-exchange. The MOF-808-TFA kinetic data, specifically the signals at 80.85 ppm (terminal –CF_3_ group from PFBS) and 58.73 ppm (−CF_3_ group in TFA), are also consistent with adsorption occurring through ion exchange. In Figure 9 we’ve plotted the loss of the PFBS signal from solution (black triangles) and the appearance of the TFA signal in solution (purple squares) over time. One can see a loss of PFBS from solution and a concomitant appearance of TFA in solution—data that is consistent with ion exchange occurring in MOF-808.

**Figure 9:**
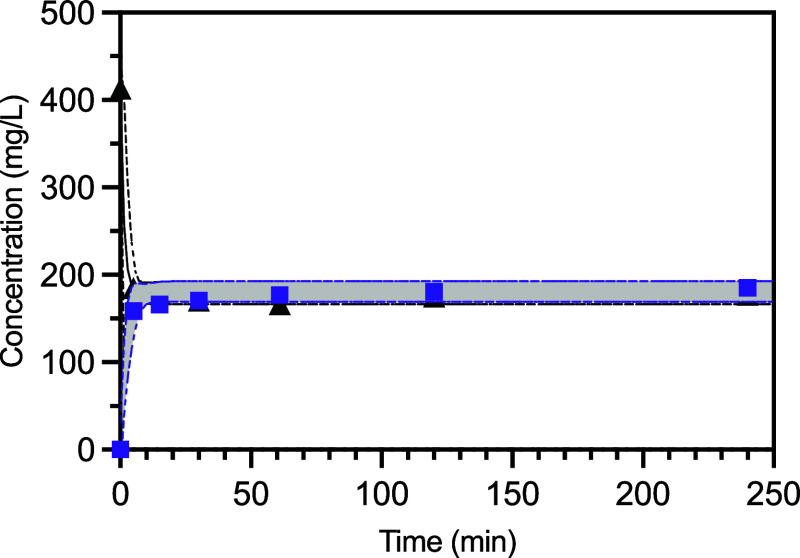
Loss of PFBS in solution (black triangles) and appearance of TFA in solution (purple squares) during PFBS adsorption in MOF-808-TFA.

Two potential ion-exchange mechanisms, inner and outer sphere, for MOF-808-AF are shown in Scheme 1. There is single-crystal X-ray evidence for outer sphere chloride ions at [Zr_6_(u_3_-O)_4_(u_3_–OH)_4_]^12+^ nodes in NU-1000,^28^ which is not surprising given the oxophilic nature of Zr^IV^.^29^ Fits of the PFBS isotherms to the Langmuir equation indicate adsorption occurs through monolayer formation at a single adsorption site. Assuming adsorption occurs at the cca’s and that there are six cca’s per [Zr_6_(u_3_-O)_4_(u_3_–OH)_4_]^12+^ node the maximum adsorption capacity for PFBS would be 1393, 1289, 1080, and 1188 mg PFBS/g MOF for MOF-808-FA, -AA, -TFA, and -AF. *q*_max_ values from the Langmuir fits to the PFBS adsorption isotherms (Figure 7) indicate that 27, 40, 67, and 70% ion-exchange occurs in MOF-808-FA, -AA, -TFA, and -AF supporting the single-site adsorption assumption. This contrasts with the work of Chang et al., who suggested that the adsorption of PFOA on MOF-808 likely occurs through a combination of hydrophobic interactions and hydrogen bonding.^30^ We are uncertain if these differences are simply due to the difference in PFAS studied (PFBS vs PFOA) or more deeply rooted in the precise composition of the MOF-808 nodes as they were not rigorously evaluated in that study. At this point in time we do not know precisely what molecular level factors are driving cca-dependent ion-exchange, however ongoing experiments in our laboratory are aimed at teasing out these details.

**Scheme 1:**
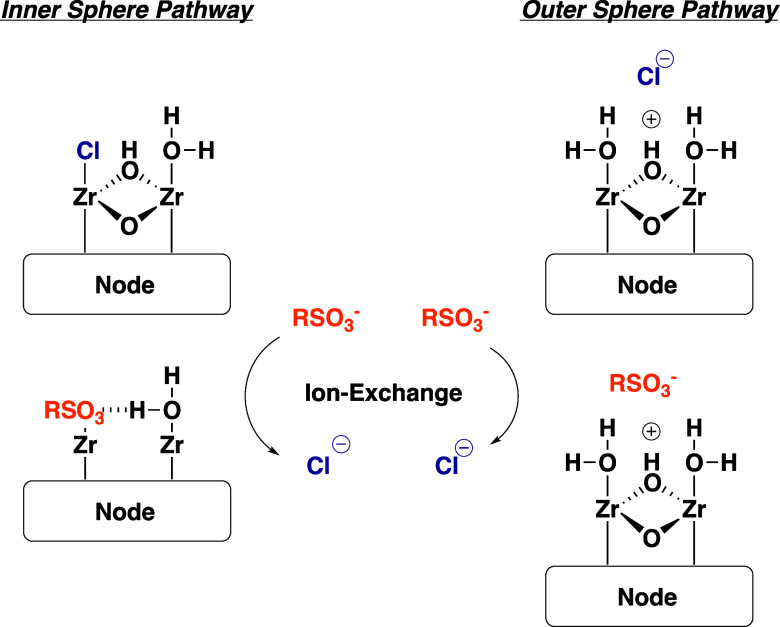
Representations of Inner Sphere (Left) and Outer Sphere (Right) Ion Exchange Mechanisms Within MOF-808

The Langmuir parameters extracted from the PFBS adsorption isotherms indicate weak interactions (*K*_L_ ranges from 1.32 × 10^–3^ to 7.72 × 10^–3^ L/mg) between PFBS and MOF-808 suggesting that ion-exchange occurs through the outer-sphere pathway. *K*_L_ values for short-chain PFAS on traditional ion-exchange resins (where inner-sphere ion exchange is commonly found) range from approximately 1–100 L/mg.^31^ Long-chain PFAS also have relatively weak interactions with MOF-808—*K*_L_ = 0.028 L/mg for PFOS on MOF-808-FA^30^ and *K*_L_ = 0.0025 L/mg and 0.0058 L/mg for PFOA on MOF-808-FA and MOF-808-TFA further supporting the outer sphere ion exchange pathway. More thorough mechanistic studies are underway to determine the precise mechanism(s) through which ion exchange occurs in MOFs with [Zr_6_(u_3_-O)_4_(u_3_–OH)_4_]^12+^ nodes.

## 5 Conclusions

MOF-808 has rapid adsorption kinetics (equilibrium reached within 50 min for all derivatives) and the highest known (up to 837 mg/g) PFBS adsorption capacity for all MOFs to date. Kinetic and cca-dependent adsorption data indicate that that cca’s play a significant role in PFBS adsorption within MOF-808. Controlling the cca’s present on MOFs with [Zr_6_(u_3_-O)_4_(u_3_–OH)_4_]^12+^ nodes will be important in optimizing and understanding PFAS adsorption within these materials. Cca-dependent adsorption, ATR, and kinetic data are all consistent with the adsorption mechanism occurring through ion exchange. Weak interactions (*K*_L_ values range from 1.32 × 10^–3^ to 7.72 × 10^–3^ L/mg) between PFBS and MOF-808 hint that ion exchange goes through an outer sphere ion exchange pathway. Further unraveling the precise mechanism(s) by which cca’s influence PFAS adsorption will help us understand how to rationally exploit cca’s to design new and improved MOFs for targeted adsorption.

## Supplementary Material



## Notes

T_1_ relaxation measurements and fitting, additional ^1^H and ^19^F­{^1^H} NMR, SEM–EDX, TGA, ATR, and PXRD characterization and fits of to the Freundlich equation can be found in the Supporting Information (PDF)
