# Supplementary material for: Influence of Charge Compensating Anions on the Adsorption of Perfluorobutanesulfonate in MOF-808
Source: ACS Omega. 2025 Sep 18;10(38):44311–20. doi: 10.1021/acsomega.5c05815 (PMC12489849; doi:10.1021/acsomega.5c05815)
Supplement: Supplementary file 1 [file ao5c05815_si_001.pdf]

Supplementary Information

Influence of Charge Compensating Anions on the Adsorption of Perfluorobutane Sulfonate in MOF-808

Jackson Mikel, Brody Berens, Grace Versnik, Kiley Wadzinski, Trevor Rottiger, Melissa Siewert, Olivia Stellpflug, Shannon C. Riha, and Joseph E. Mondloch*

University of Wisconsin–Stevens Point, Stevens Point, WI 54481, United States.

Table of Contents

Content	Page(s)
Quantitative NMR Spectra Collection	S2
PXRD and Nitrogen Adsorption Characterization of MOF-808 Derivatives	S3
Determination of Charge Compensating Anions via NMR	S4–S8
Determination of Charge Compensating Anions via EDX	S9
TGA Data	S10–S12
Full ATR IR Spectra	S13
Post PFBS Adsorption PXRD	S14
Freundlich Fits to PFBS Adsorption Isotherms	S15
Charge Compensating Anion Control Reactions	S16–S18

Quantitative NMR Spectra Collection

To ensure that our ^{19}F NMR measurements were quantitative, T1 inversion recovery experiments were performed. An example T1 inversion recovery for a PFBS and 2,6-TFMBA mixture is shown in Figure S1. The signal at -80.7 ppm comes from the $-CF_3$ resonances of 2,6-TFMBA, while the signal at -59.6 ppm is the terminal $-CF_3$ group of PFBS.

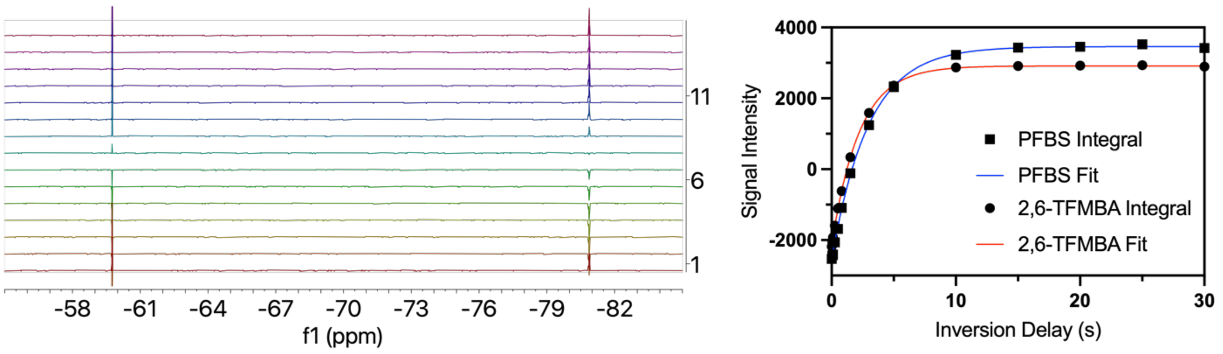

Figure S1. Shown on the left are T1 relaxation delay measurements for 2,6-TFMBA (-59.6 ppm) and the terminal $-CF_3$ group of PFBS (-80.7 ppm). Shown on the right are fits of equation S1 to the 2,6-TFMBA and PFBS signals respectively.

The three-parameter equation, equation S1, was utilized to fit the inversion delay data shown in Figure S1. In equation S1 T1 is equal to 1/G and the relaxation delays utilized for quantitative NMR calculations were set to at least 5x the longest measured T1. Fitting parameters for both 2,6-TFMBA and the terminal $-CF_3$ of PFBS are shown in Table S1.

$$y = B + F \times \exp^{(-x*G)} \quad \text{(S1)}$$

Table S1. Best fit data for T1 inversion delay experiment using equation S1.

**2,6-TFMBA Fit (-59.6 ppm)**	**$-CF_3$ PFBS Fit (-80.7 ppm)**
B = 2912	B = 3461
F = -5062	F = -6014
G = 0.447	G = 0.336
T1 = 1/G = 2.24 s	T1 = 1/G = 2.98 s
R^2 = 0.9999	R^2 = 0.9998
Required Delay = 11.2 s	Required Delay = 14.9 s

<u>*PXRD and Surface Area Characterization of MOF-808 Derivatives*</u>

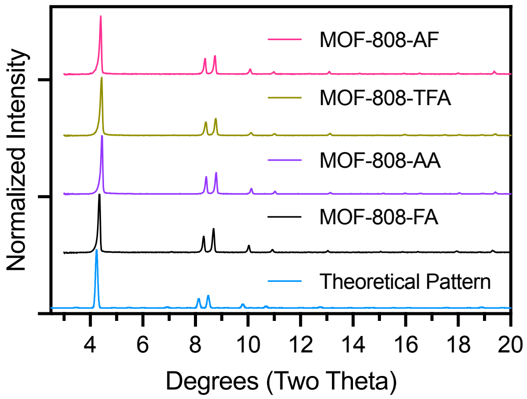

Figure S2. Powder X-ray diffraction patterns of as synthesized MOF-808-FA (black), MOF-808-AA (purple), MOF-808-TFA (gold), and MOF-808-AF (pink) in comparison to the theoretical pattern of MOF-808-FA (blue).

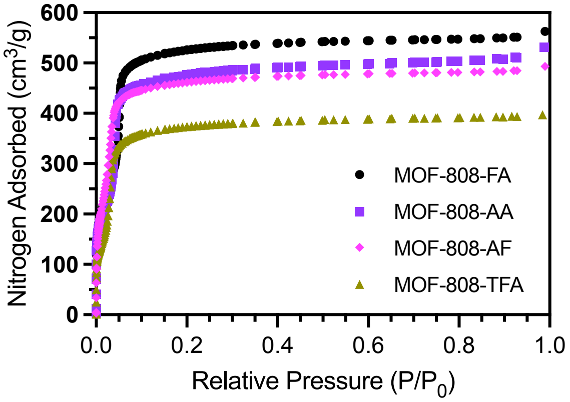

Figure S3. Nitrogen adsorption isotherms for MOF-808-FA (black circles), MOF-808-AA (purple squares), MOF-808-TFA (gold triangles), and MOF-808-AF (pink diamonds).

Table S1. Pore volume and BET surface area for MOF-808-FA, MOF-808-AA, MOF-808-TFA, and MOF-808-AF.

MOF	This Work Pore Volume (cm^3/g)	This Work BET Surface Area (m^2/g)	Literature Pore Volume (cm^3/g)	Literature BET Surface Area (m^2/g)	Reference
MOF-808-FA	0.85	2133	2130	0.86	**1**
MOF-808-AA	0.79	1875	1850	0.76	**1**
MOF-808-TFA	0.61	1461	1490	0.62	**1**
MOF-808-AF	0.76	1808	1955	0.78	**1**

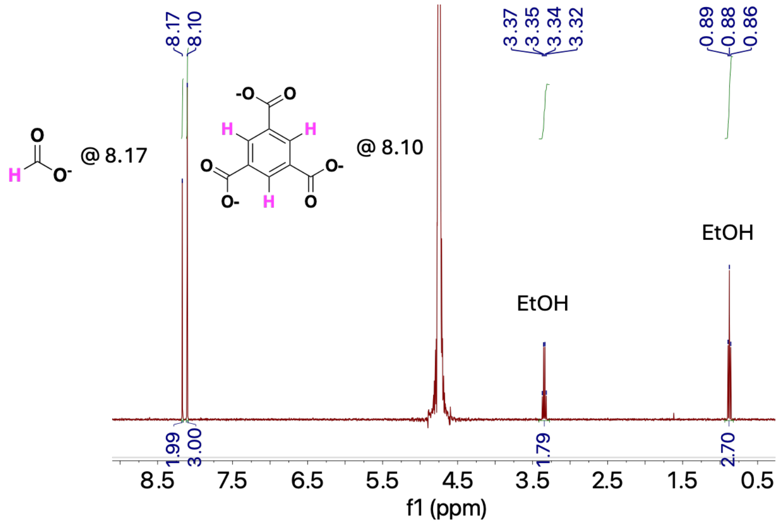

Figure S4. Quantification of formate in MOF-808-FA before PFBS adsorption.

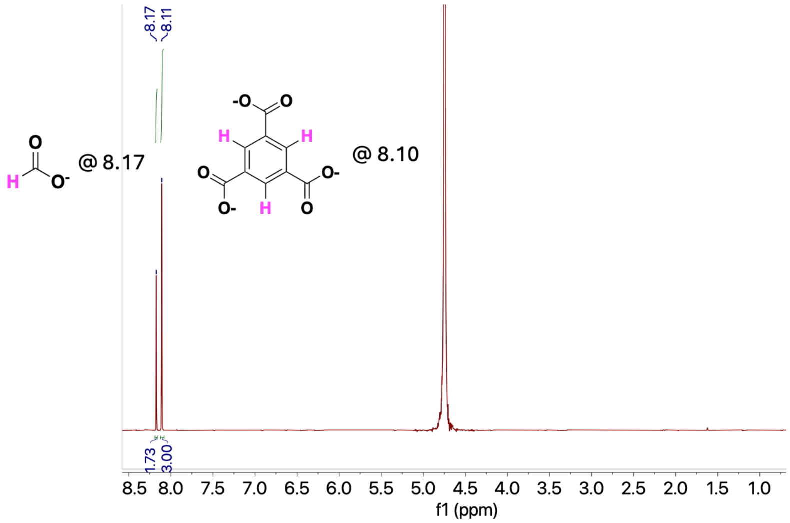

Figure S5. Quantification of formate in MOF-808-FA after PFBS adsorption.

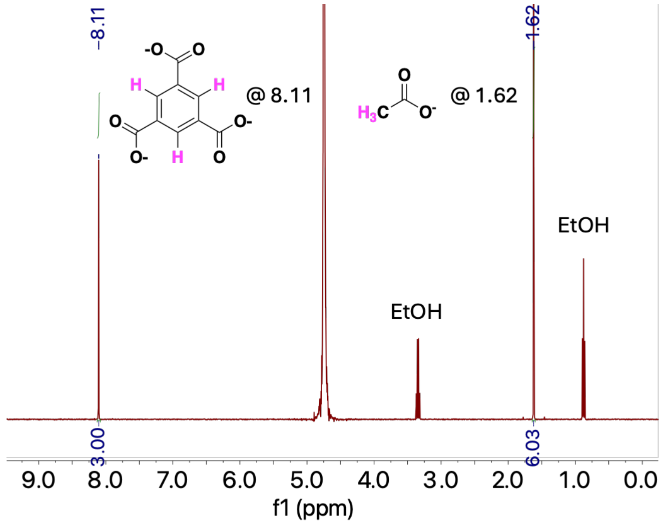

Figure S6. Quantification of acetate in MOF-808-AA before PFBS adsorption.

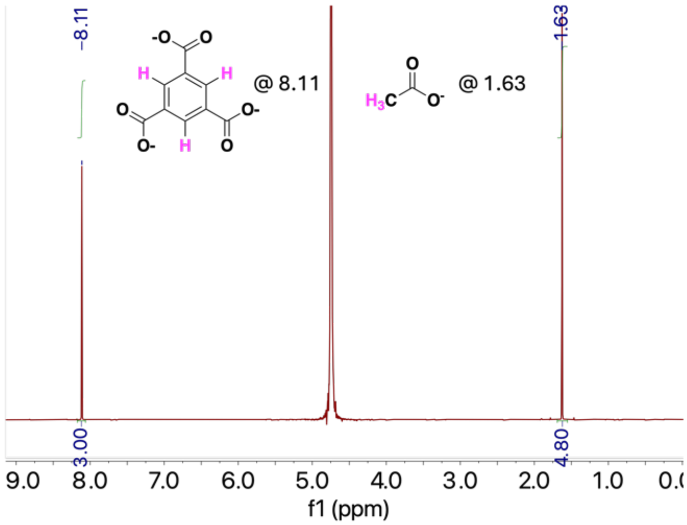

Figure S7. Quantification of acetate in MOF-808-AA after PFBS adsorption.

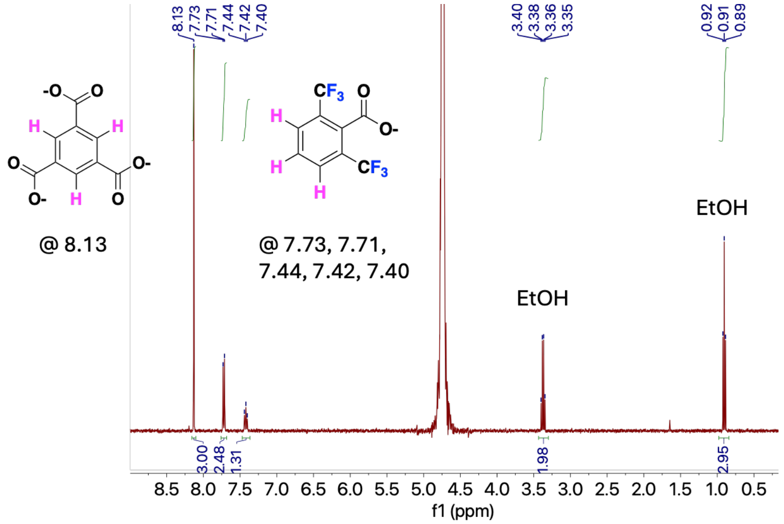

Figure S8. Quantification of trifluoroacetate via ^{1}H NMR in MOF-808-TFA before PFBS adsorption.

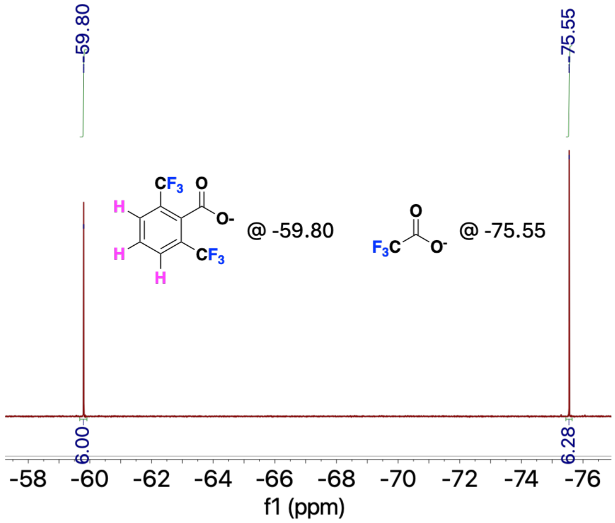

Figure S9. Quantification of trifluoroacetate (TFA) via $^{19}F\{^{1}H\}$ NMR in MOF-808-TFA before PFBS adsorption.

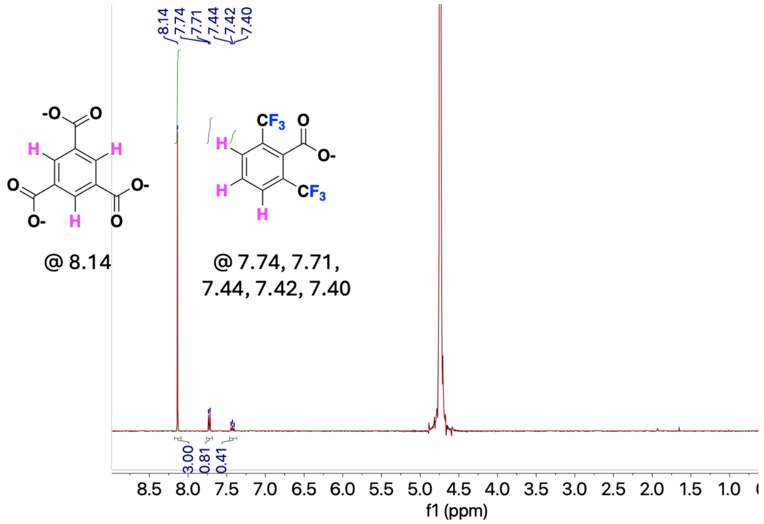

Figure S10. Quantification of trifluoroacetate via ^{1}H NMR in MOF-808-TFA after PFBS adsorption.

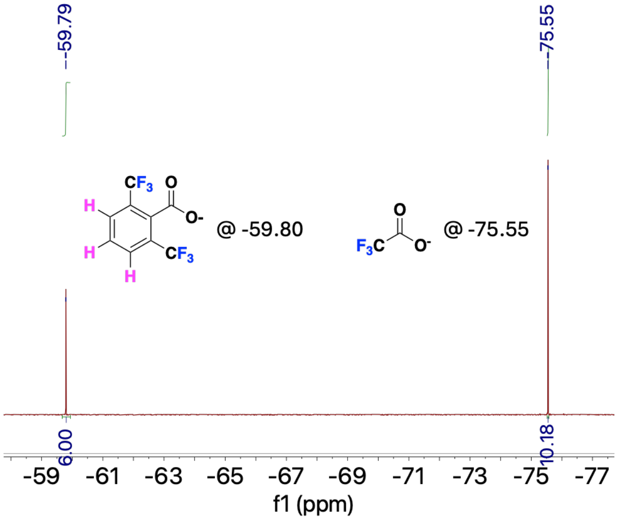

Figure S11. Quantification of trifluoroacetate via $^{19}F\{^{1}H\}$ NMR in MOF-808-TFA after PFBS adsorption.

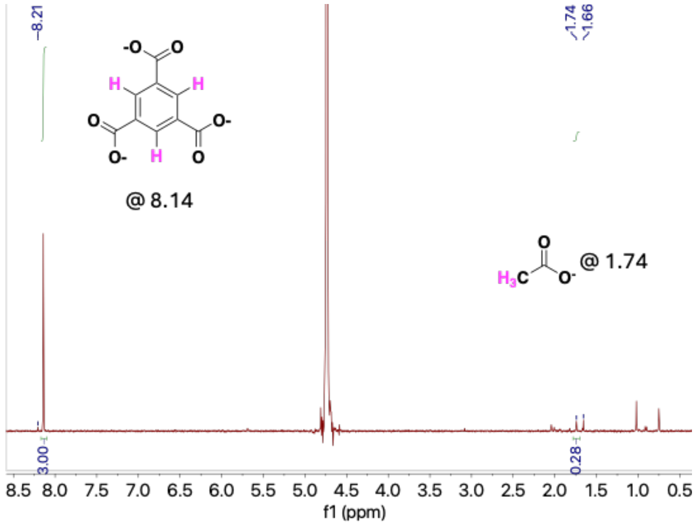

Figure S12. Quantification of acetate in MOF-808-AF before PFBS adsorption.

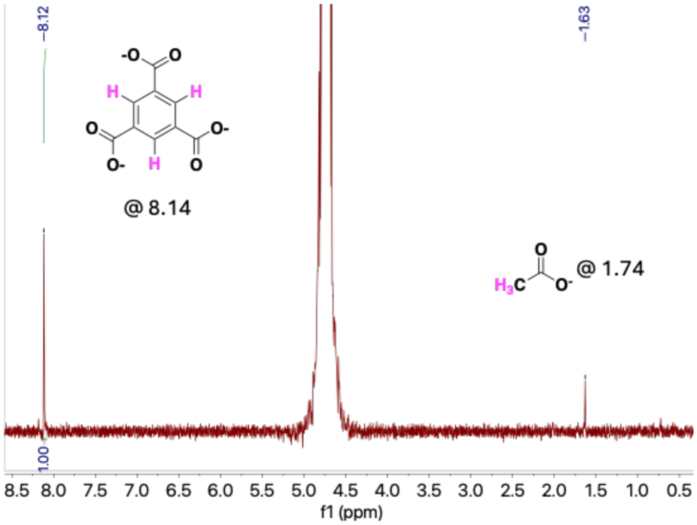

Figure S13. Quantification of acetate in MOF-808-AF after PFBS adsorption.

<u>*Characterization of Chloride Charge Compensating Anions via EDX Analysis*</u>

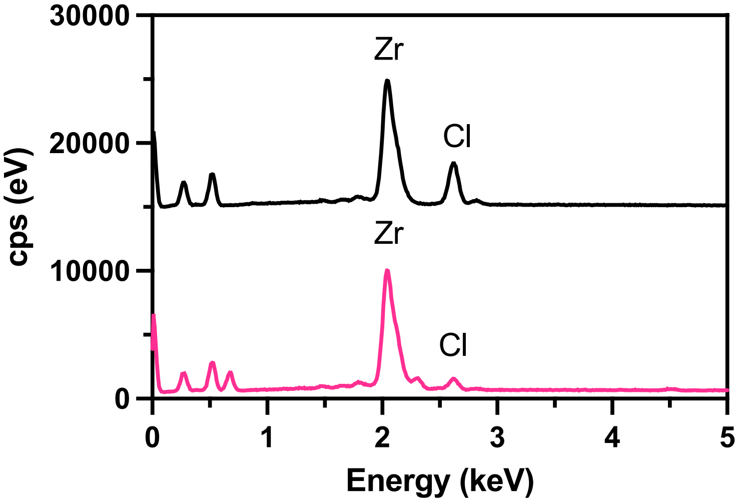

Figure S14. An example EDX spectra showing the location of Zr and Cl used to calculate the amount of chloride before (black) and after (pink) PFBS adsorption in Table 1 of the main text. This data is from MOF-808-AF.

TGA Data

The quantification of cca's shown in Table 1 of the main text assumes that defects are not present in our MOF-808 derivatives. More specifically the quantity of FA, AA, and TFA was determined assuming two btc linkers per $[Zr_6(u_3\text{-}O)_4(u_3\text{-}OH)_4]^{12+}$ node and the quantity of chloride was determined assuming six zirconium ions per $[Zr_6(u_3\text{-}O)_4(u_3\text{-}OH)_4]^{12+}$ node. To ensure that these are acceptable assumptions we turned to thermal gravimetric analysis (TGA) which has been used extensively to probe for defects in MOFs containing $[Zr_6(u_3\text{-}O)_4(u_3\text{-}OH)_4]^{12+}$ nodes. This analysis assumes complete conversion to six equivalents of ZrO_2 on heating to high temperature and that the associated mass loss is due to loss of water, cca's, and linkers from the framework via desorption or decomposition. The weight percent at the end of the thermal event should be consistent with six equivalents of ZrO_2 and mass losses lower than expected are taken as evidence for linker defects which in turn would affect the number of cca's calculated in Table 1. An example of the reaction stoichiometry for an idealized MOF-808-FA derivative is shown in equation S2.

$$Zr_6(u_3\text{-}O)_4(u_3\text{-}OH)_4(btc)_2(FA)_6\ (s) + 21\ O_2\ (g) \rightarrow 6\ ZrO_2\ (s) + 24\ CO_2\ (g) + 8\ H_2O\ (g) \quad \text{(S2)}$$

The theoretical weight percent of six equivalents of ZrO_2 can be calculated from this equation and the weight percent loss (expected from combustion of the linkers, cca's and $EtOH/EtO^-$) can be obtained by subtracting that value from 100%. In the Table below we've calculated the theoretical weight percent of six equiv of ZrO_2 (cell highlighted in green) based on the empirical formulas determined from our NMR and SEM-EDX data (*i.e.*, the moocarboxylate, ethanol, and chloride data). Consideration of all this data yielded final ZrO_2 weight percentages that were lower than expected indicating some unaccounted-for mass. Therefore terminal water molecules were included when larger than expected mass losses were observed. This allows total charge compensating to occur either as $EtOH/EtO^-$ or H_2O/OH^- pairs and a proton would have to be removed somewhere from this analysis. Fortunately, the mass of a proton is small and has a negligible effect on the calculated weight percentages reported below. Comparison of the theoretical and observed mass is shown in Table S3 below. The experimental TGA data is shown in Figure S15.

Table S2. Calculated theoretical weight percentages of six equivalents of ZrO_2 for the empirical formulas for MOF-808 found herein.

MOF-808-FA	Node	Node	Node	Linker	Carboxylate	Terminal	Chloride	EtOH	ZrO2
Composition	Zr6	O6	H2O	C9H3O6	CO2H	H2O	Cl	CH3CH2OH	ZrO2
Formula Weight (amu)	547.34	95.994	18.015	207.12	45.02	18.015	35.453	46.068	123.218
Number	1	1	2	2	4	4	0.8	1.8	6
Total Weight	547.34	95.994	36.03	414.24	180.08	72.06	28.3624	82.9224	739.308
Weight Percent	37.57	6.59	2.47	28.43	12.36	4.95	1.95	5.69	50.74
MOF-808-AA	Node	Node	Node	Linker	Carboxylate	Terminal	Chloride	EtOH	ZrO2
Composition	Zr6	O6	H2O	C9H3O6	CO2CH3	H2O	Cl	CH3CH2OH	ZrO2
Formula Weight (amu)	547.344	95.994	18.015	207.12	59.04	18.015	35.453	46.068	123.218
Number	1	1	2	2	4	4	1.6	2.5	6
Total Weight	547.344	95.994	36.03	414.24	236.16	72.06	56.7248	115.17	739.308
Weight Percent	34.78	6.10	2.29	26.32	15.01	4.58	3.60	7.32	46.98
MOF-808-TFA	Node	Node	Node	Linker	Carboxylate	Terminal	Chloride	EtOH	ZrO2
Composition	Zr6	O6	H2O	C9H3O6	CO2CF3	H2O	Cl	CH3CH2OH	ZrO2
Formula Weight (amu)	547.344	95.994	18.015	207.12	113.02	18.015	35.453	46.068	123.218
Number	1	1	2	2	5.2	6	0	1.95	6
Total Weight	547.344	95.994	36.03	414.24	587.704	108.09	0	89.8326	739.308
Weight Percent	29.13	5.11	1.92	22.04	31.27	5.75	0.00	4.78	39.34
MOF-808-AF	Node	Node	Node	Linker	Carboxylate	Terminal	Chloride	EtOH	ZrO2
Composition	Zr6	O6	H2O	C9H3O6	CO2CH3	H2O	Cl	CH3CH2OH	ZrO2
Formula Weight (amu)	547.344	95.994	18.015	207.12	59.04	18.015	35.453	46.068	123.218
Number	1	1	2	2	0.2	26	3.8	0	6
Total Weight	547.344	95.994	36.03	414.24	11.808	468.39	134.7214	0	739.308
Weight Percent	32.04	5.62	2.11	24.25	0.69	27.41	7.89	0.00	43.27

Table S3. Theoretical and experimental comparison of the final weight percent observed in the TGA experiments.

MOF	Empirical Formula	ZrO_2 Theoretical	ZrO_2 Observed
MOF-808-FA	$Zr_6(u_3\text{-}O)_4(u_3OH)_4(btc)_2(FA)_4(Cl)_{0.8}(H_2O)_4(EtOH)_{1.8}$	50.74	50.67
MOF-808-AA	$Zr_6(u_3\text{-}O)_4(u_3OH)_4(btc)_2(AA)_4(Cl)_{1.6}(H_2O)_4(EtOH)_{2.5}$	46.98	47.41
MOF-808-TFA	$Zr_6(u_3\text{-}O)_4(u_3OH)_4(btc)_2(TFA)_{5.2}(H_2O)_6(EtOH)_{2.0}$	39.34	38.69
MOF-808-AF	$Zr_6(u_3\text{-}O)_4(u_3OH)_4(btc)_2(AA)_{0.2}(Cl)_{3.8}(OH/H_2O)_{26}$	43.27	43.14

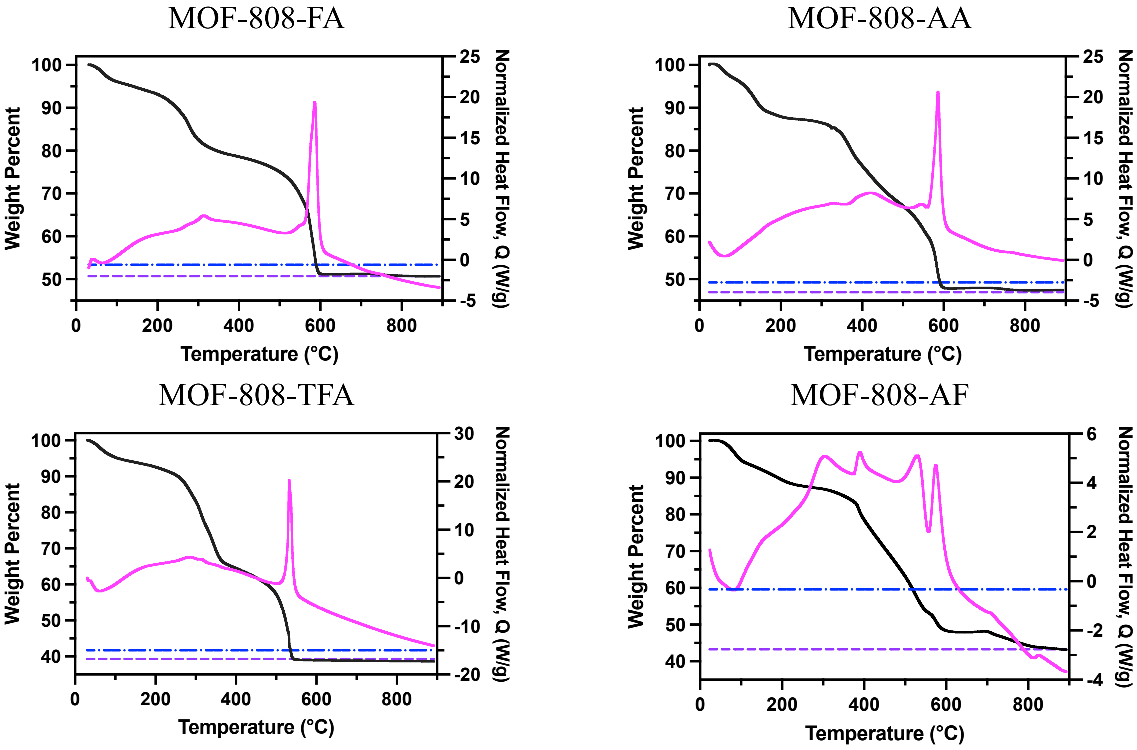

Figure S15. TGA and DTA curves for MOF-808-FA, -AA, -TFA, and -AF. The dashed blue line is the expected final weight percent for 6 ZrO_2 without water adsorbed onto the MOF, while the dashed purple line is the final weight percent for 6 ZrO_2 with water adsorbed onto the MOF as detailed in the main text.

ATR IR Data

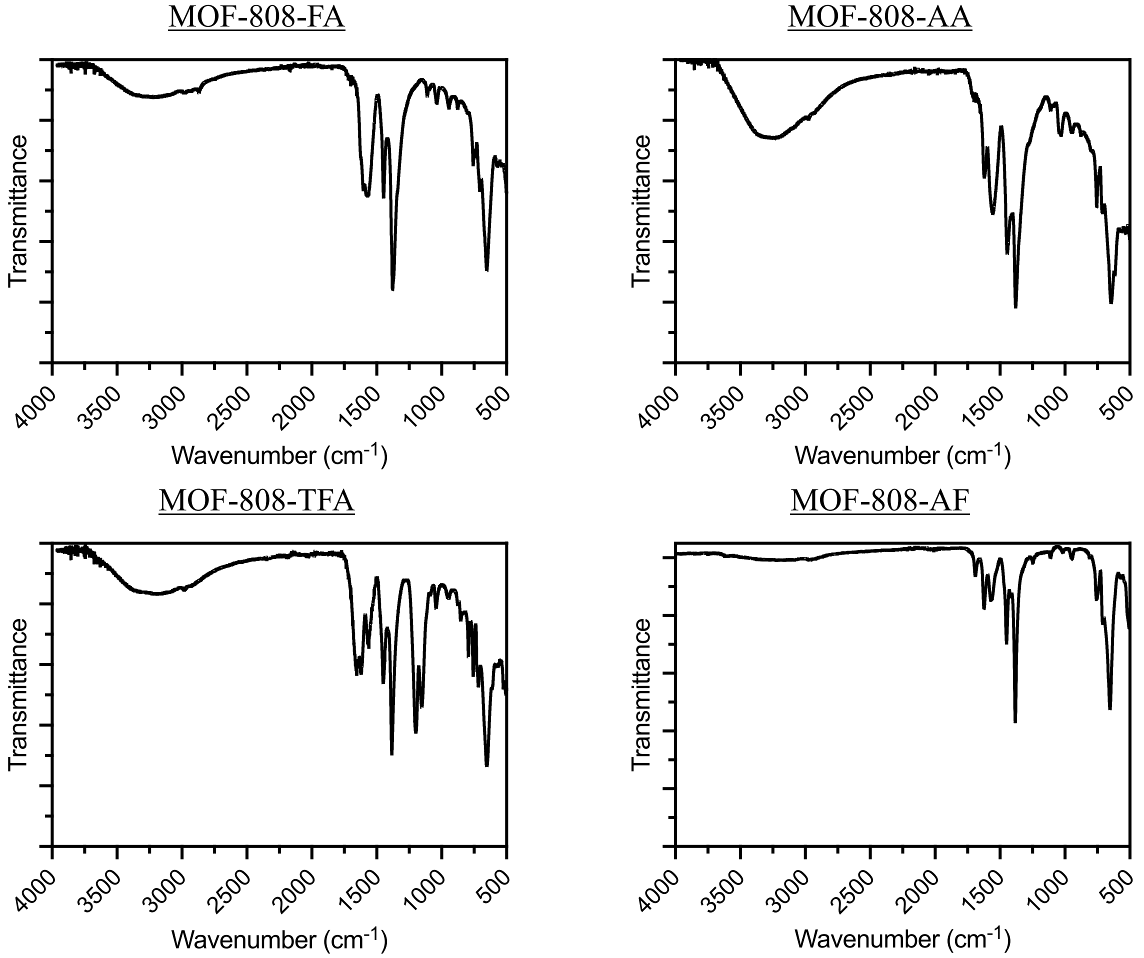

Figure S16. Full ATR IR spectra for MOF-808-FA, -AA, -TFA, and -AF.

PXRD Post PFBS Adsorption

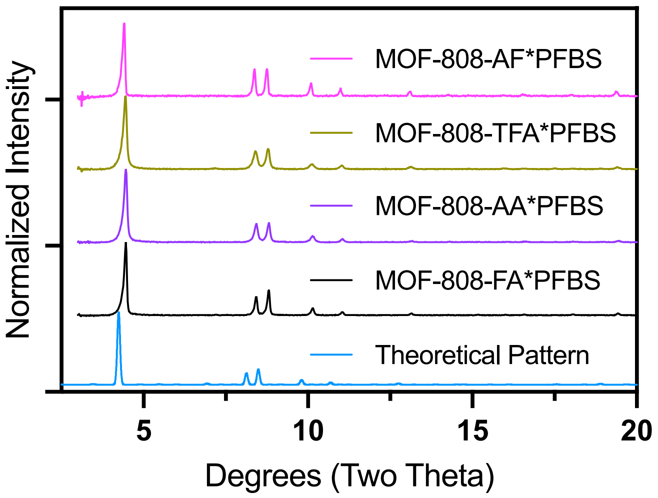

Figure S17. MOF-808-FA, -AA, -TFA, and -AF PXRD patterns post PFBS adsorption.

PFBS Isotherms and Fits to the Freundlich Equation

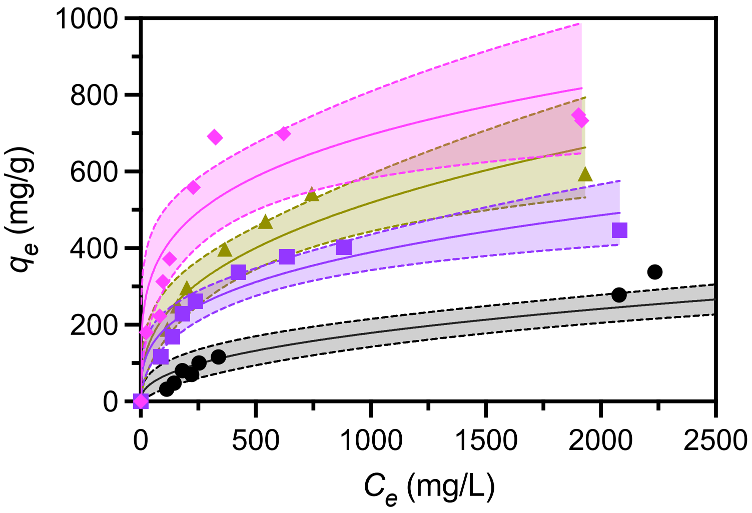

Figure S18. PFBS adsorption isotherms for MOF-808-FA (black circles), MOF-808-AA (purple squares), MOF-808-TFA (gold triangles), and MOF-808-AF (pink diamonds). The solid lines represent fits to the Freundlich equation (eq S4) and the bands represent 95% confidence intervals for the Freundlich fits.

Table S4. Summary of the quantity of cca's and EtOH in each MOF-808 derivative in the absence of PFBS (*i.e.*, after a standard conditions adsorption experiment in just DI water).

MOF	Carboxylate	Chloride	EtOH
MOF-808-FA	3.7	0.4	0.0
MOF-808-AA	3.8	1.2	0.0
MOF-808-TFA	4.0	0.0	0.0
MOF-808-AF	0.1	2.8	0.0

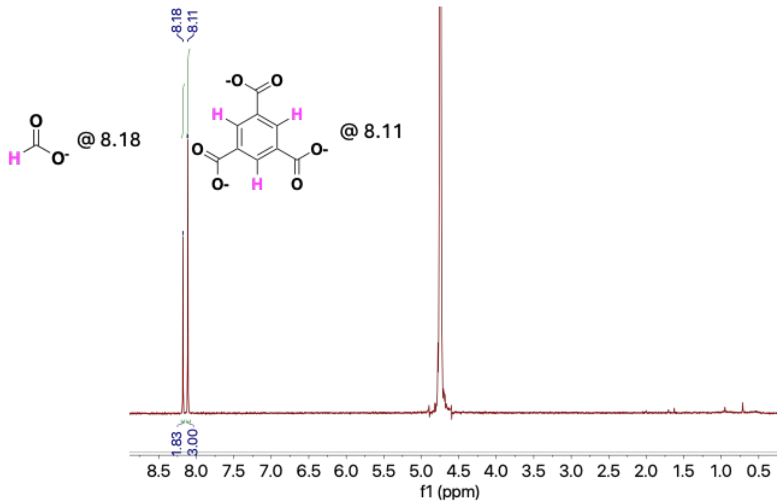

Figure S19. Quantification of formate in MOF-808-FA in the absence of PFBS after a standard conditions reaction.

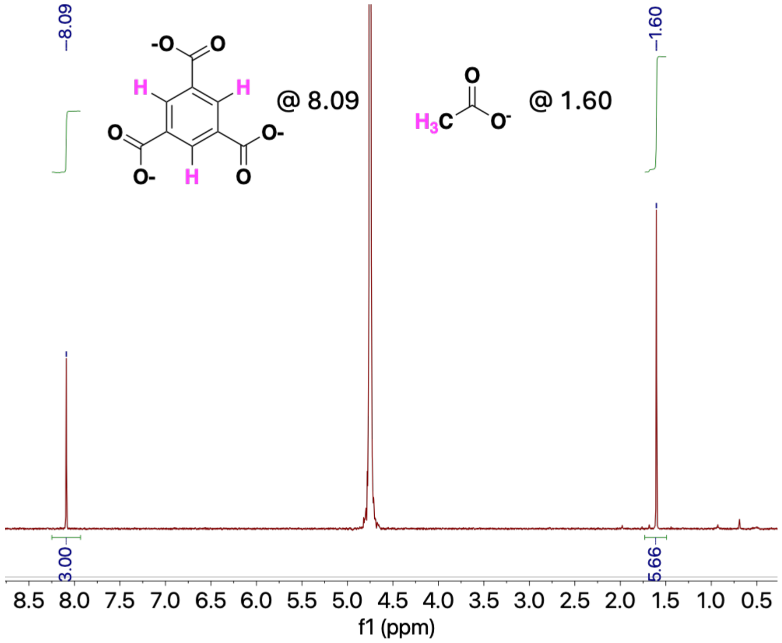

Figure S20. Quantification of acetate in MOF-808-AA in the absence of PFBS after a standard conditions reaction.

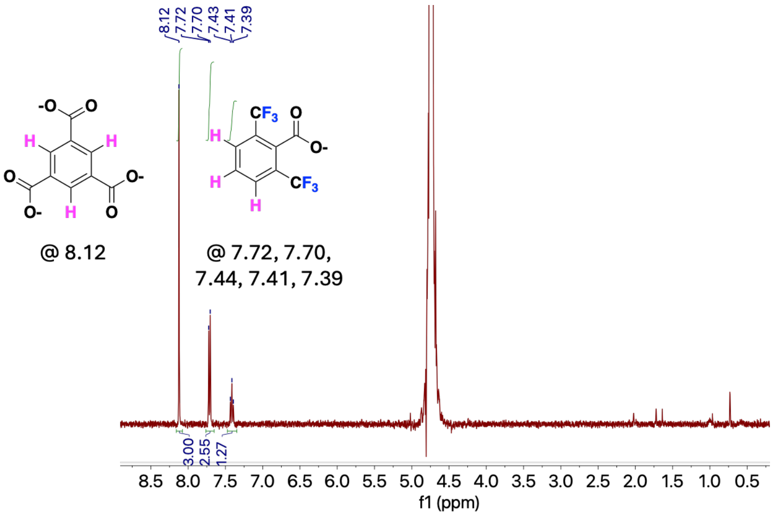

Figure S21. ^{1}H NMR quantification of trifluoroacetate in MOF-808-TFA in the absence of PFBS after a standard conditions reaction.

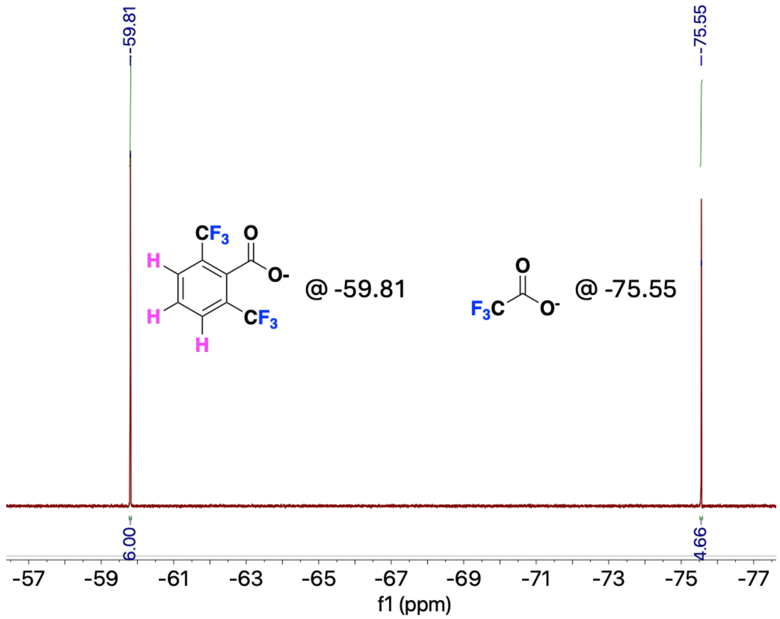

Figure S22. ^{19}F NMR quantification of trifluoroacetate in MOF-808-TFA in the absence of PFBS after a standard conditions reaction.

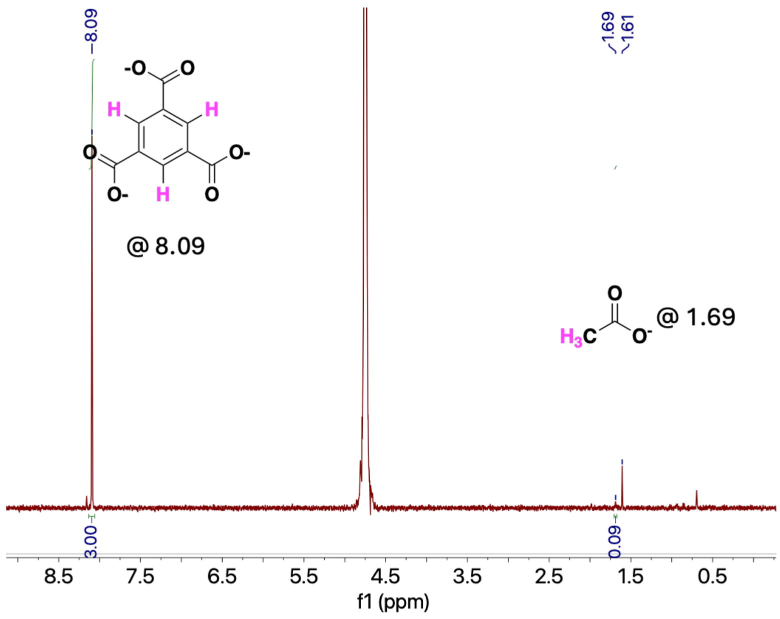

Figure S23. Quantification of acetate in MOF-808-AF in the absence of PFBS after a standard conditions reaction.
